# Assessing similarity to primary tissue and cortical layer identity in induced pluripotent stem cell-derived cortical neurons through single-cell transcriptomics

**DOI:** 10.1093/hmg/ddv637

**Published:** 2016-01-05

**Authors:** Adam E. Handel, Satyan Chintawar, Tatjana Lalic, Emma Whiteley, Jane Vowles, Alice Giustacchini, Karene Argoud, Paul Sopp, Mahito Nakanishi, Rory Bowden, Sally Cowley, Sarah Newey, Colin Akerman, Chris P. Ponting, M. Zameel Cader

**Affiliations:** 1 Department of Physiology, Anatomy and Genetics, University of Oxford, Oxford, Oxfordshire OX1 3QX, UK,; 2 Weatherall Institute of Molecular Medicine, University of Oxford, Oxford, Oxfordshire OX3 9DS, UK,; 3 Department of Pharmacology, University of Oxford, Oxford, Oxfordshire OX1 3QT, UK,; 4 Dunn School of Pathology, University of Oxford, Oxford, Oxfordshire OX1 3RE, UK,; 5 Wellcome Trust Centre for Human Genetics, University of Oxford, Oxford, Oxfordshire OX3 7BN and; 6 Research Center for Stem Cell Engineering, National Institute of Advanced Industrial Science and Technology, Tsukuba, Ibaraki, Japan

## Abstract

Induced pluripotent stem cell (iPSC)-derived cortical neurons potentially present a powerful new model to understand corticogenesis and neurological disease. Previous work has established that differentiation protocols can produce cortical neurons, but little has been done to characterize these at cellular resolution. In particular, it is unclear to what extent *in vitro* two-dimensional, relatively disordered culture conditions recapitulate the development of *in vivo* cortical layer identity. Single-cell multiplex reverse transcriptase-quantitative polymerase chain reaction (RT-qPCR) was used to interrogate the expression of genes previously implicated in cortical layer or phenotypic identity in individual cells. Totally, 93.6% of single cells derived from iPSCs expressed genes indicative of neuronal identity. High proportions of single neurons derived from iPSCs expressed glutamatergic receptors and synaptic genes. And, 68.4% of iPSC-derived neurons expressing at least one layer marker could be assigned to a laminar identity using canonical cortical layer marker genes. We compared single-cell RNA-seq of our iPSC-derived neurons to available single-cell RNA-seq data from human fetal and adult brain and found that iPSC-derived cortical neurons closely resembled primary fetal brain cells. Unexpectedly, a subpopulation of iPSC-derived neurons co-expressed canonical fetal deep and upper cortical layer markers. However, this appeared to be concordant with data from primary cells. Our results therefore provide reassurance that iPSC-derived cortical neurons are highly similar to primary cortical neurons at the level of single cells but suggest that current layer markers, although effective, may not be able to disambiguate cortical layer identity in all cells.

## Introduction

Investigating the cellular basis of neurological diseases, especially those affecting the central nervous system (CNS), is rendered particularly challenging by the inaccessibility of the tissues involved. Induced pluripotent stem cell (iPSC)-based models have the potential to allow *in vitro* investigation of these tissues in human samples from patients affected by such diseases and, importantly, how disease progresses over time (1). Protocols have been developed capable of generating cortical cells from human iPSCs, which appear to adopt specific cortical layer identities and develop functional synapses (2–6).

Most transcriptomic studies of iPSC-derived cortical neurons have examined expression in samples pooled from a whole population of cells so would miss potential cell type-specific or layer-specific effects (7,8). The development of single-cell gene expression platforms, such as microfluidic chips, as well as evolving chip-free single-cell RNA-seq technologies, make such studies a viable method to investigate iPSC-derived cortical neuron cultures at single-cell resolution (9,10). This has the advantage that the relative abundance of different cell types may be discerned, and so comparisons between iPSC-derived and primary tissues can be made at the level of individual cells.

A core set of cortical layer markers has been used within the stem cell research community to establish the presence of neurons with different layer identities in iPSC-derived cortical neuronal cultures (2,4,11). However, many of these markers were inferred from studies of mouse brain or immunohistochemistry of human fetal brain, so the robustness of such markers in assigning layer identity to single neurons by single-cell transcriptomics approaches is unknown (12,13).

The degree of heterogeneity present in cortical neurons derived from iPSCs is a critically important aspect of *in vitro* models to understand. Layer-specific and phenotypic cellular identity is particularly relevant prior to applying such models to address disease-specific hypotheses.

Cortical neurons derived from iPSCs using such methods have been used to study a wide variety of neurodevelopmental and neurodegenerative conditions, and recapitulate disease-relevant phenotypes (1). In the case of Alzheimer's disease, iPSC-derived cortical neurons displayed aberrant Aβ secretion and tau phosphorylation (8,14). iPSC lines from autism spectrum disorder patients showed abnormalities in deep cortical layer formation and resulted in overproduction of GABAergic interneurons (11,15). Studying the effect of disease pathology at a single-cell level is an attractive approach as it may allow identification of cellular processes that cause cell type or layer-specific vulnerability (16).

Here, we used single-cell transcriptomic methodologies to investigate the extent to which iPSC-derived cortical cells express key neuronal genes relevant to cortical function. We also sought to examine whether iPSC neurons recapitulate normal cortical layer identity and to thereby assess the applicability of widely used cortical layer markers to the single-cell transcriptome.

## Results

### Single-cell RT-qPCR neuronal identity

We generated cortical neurons using a well-established protocol with small molecule dual SMAD inhibition for neural induction followed by plating of neuroepithelial cells for final differentiation (2).

**Figure 1.**
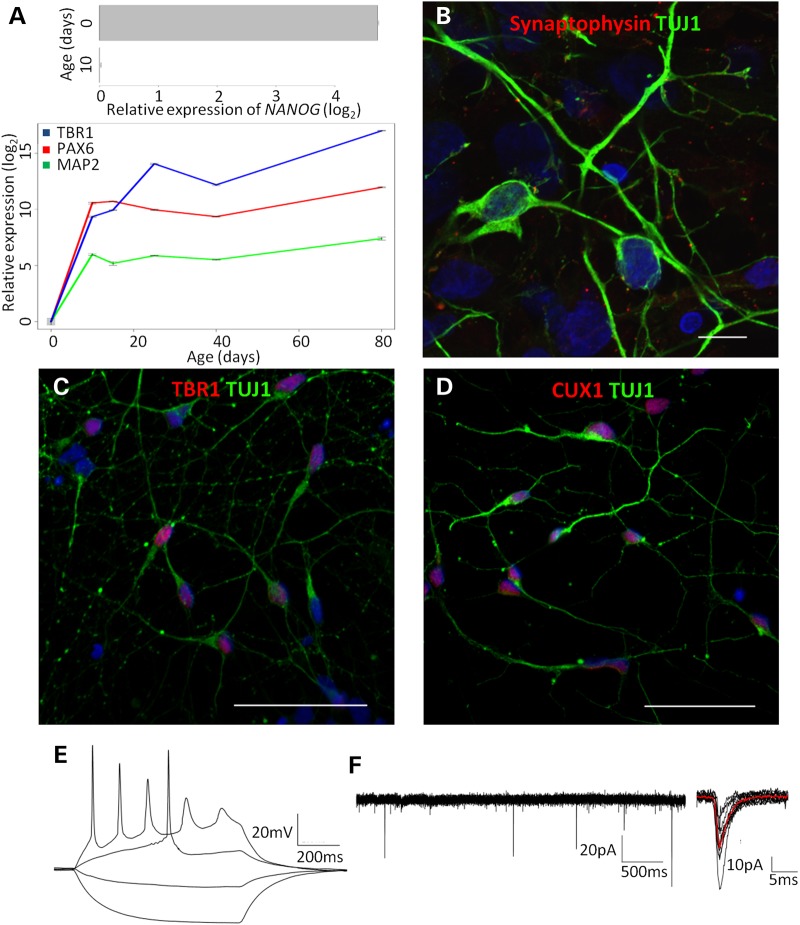
Validation of cortical neuronal phenotype. (**A**) RT-qPCR showing a reduction in *NANOG* expression (top) and increased expression of cortical identity markers (bottom) following neural induction in AH017-7. Error bars show the standard deviation from technical triplicates. Immunofluorescence microscopy for: (**B**) TUJ1 (green) and Synaptophysin (red), scale bar = 10 μm; (**C**) TUJ1 (green) and TBR1 (red), scale bar = 50 μm; (**D**) TUJ1 (green) and CUX1 (red), scale bar = 50 μm; all images are also co-stained with DAPI (blue). (**E**) Repetitive firing evoked by current clamp protocol. (**F**) Spontaneous electrical activity.

Over the course of neuronal differentiation, cultures showed the expected decrease in expression of pluripotency genes and increased expression of neuronal genes (Fig. 1A). Staining of iPSC-derived cortical neurons showed the presence of synaptic markers, the deep layer marker TBR1 and the upper layer marker CUX1 (Fig. 1B–D). Neurons demonstrated repetitive firing in response to depolarization and spontaneous synaptic activity (Fig. 1E and F), indicating functional maturation.

The use of iPSC neurons for disease modelling and drug screening requires the production of cells emulating the cell types involved in the disease processes. We characterized the identity of cells produced by this protocol by performing single-cell analysis on three iPSC lines (iPS-AH017-3, iPS-AH017-7 and iPS-NHDF-1) following cortical differentiation for at least 81 days (Supplementary Material, Table S1). Cells were dissociated into a single-cell suspension, and single cells (178 cells from AH017-3, 153 from AH017-7 and 75 from NHDF1) were sorted into polymerase chain reaction (PCR) plates using fluorescence-activated cell sorting (FACS) (Supplementary Material, Fig. S2). Multiplex single-cell reverse transcriptase-quantitative polymerase chain reaction (RT-qPCR) was carried out on 96 genes previously implicated as important for neuronal function or cortical layer identity, housekeeping genes and negative control genes (2,12,13,17–19).

**Figure 2.**
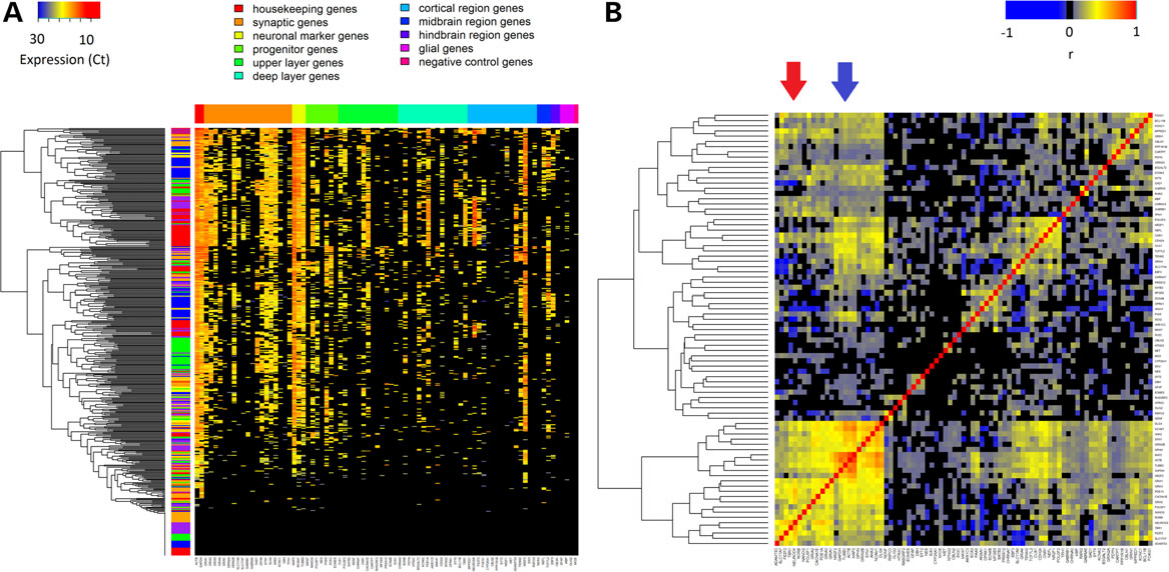
Single-cell RT-qPCR data. (**A**) Heat map of single-cell gene expression data. Cells are shown in rows and genes in columns. The vertical colour key beside the dendrogram indicates the experiment [red = AH017-3 (Repeat 1), blue = AH017-7, green = NHDF1, yellow = AH017-3 (Repeat 2), purple = AH017-3 (Repeat 3) and orange = AH017-7 (Repeat 2)]. The horizontal colour key indicates the functional category of genes. Cells are clustered by Euclidean distance. (**B**) Co-expression heat map of single-cell gene expression data. The shading represents the magnitude of the Pearson correlation coefficient. Clusters of genes relevant to neuronal function (blue) and neocortical identity (red) are indicated by arrows. Genes not detected in bulk RT-qPCR samples were removed prior to analysis.

Of the 478 wells into which a single cell had been sorted, 406 (85.3%) wells were positive for expression of the housekeeper gene *GAPDH*. Of these, 380 (93.6%) expressed markers of neuronal identity (*MAP2*, *NCAM1* or *TUBB3*) (Supplementary Material, Table S1). The majority of these also expressed genes related to glutamatergic synapse function (e.g. *GRIA1* was detected in 63.9% of neurons, *DLG4* in 70.3% and *SYN1* in 67.6%). A small proportion (23.9%) of neurons expressed *GAD1*, suggesting the presence of GABAergic inhibitory neurons. This marker was inversely correlated with *SLC17A7*, a marker of excitatory identity (*r* = −0.10, *P* = 0.03). Almost no cells had any detectable expression of glia-specific genes (*GFAP* 1.0%, *OLIG2* 0.2%). A heat map of all single-cell RT-qPCR data is shown in Figure 2A. Co-expression analysis showed clusters associated with synaptic function and neuronal identity (Fig. 2B). These included clusters containing multiple neuronal markers (e.g. *MAP2* and *NCAM1*), synaptic genes (e.g. *DLG4* and *ANK2*) and NMDA receptor genes (*GRIN2B*), with another cluster containing occipital lobe neocortical identity genes (*FEZF2*, *NEUROD6* and *ADAMTS3*) and AMPA receptor genes (*GRIA1*, *GRIA2* and *GRIA3*). We assessed whether there were any differences in the cellular composition of cultures aged between 81 days (*n* = 109) and 180 days (*n* = 69) in AH017-3 iPSC-derived cortical neurons and found no significant difference (*P* = 0.44).

There was good correlation between bulk and pooled single-cell gene expression (AH017-3: *r* = 0.74, *P* < 0.0001; AH017-7: *r* = 0.78, *P* < 0.0001; NHDF1: *r* = 0.72, *P* < 0.0001; Supplementary Material, Fig. S3).

### Comparison with single-cell RNA-seq

**Figure 3.**
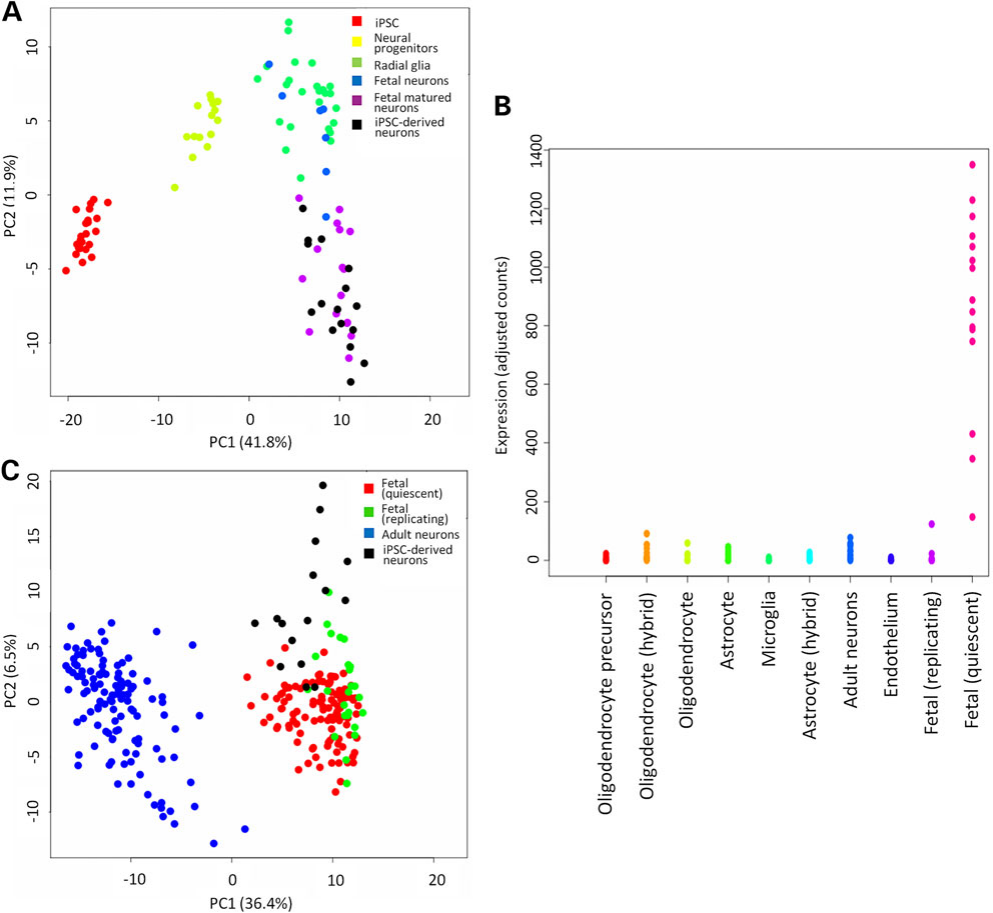
Single-cell RNA-seq in iPSC-derived cortical neurons. (**A**) PCA of single-cell RNA-seq on iPSCs (red), neural progenitors (yellow), fetal radial glia (green), fetal newborn cortical neurons (21-week post-conception; blue), fetal matured cortical neurons (21-week post-conception plus 3-week culture; purple) and iPSC-derived cortical neurons (black) (20). (**B**) Mean expression levels of genes demonstrated by Darmanis *et al.* to distinguish between different types of adult and fetal cells within the CNS (21). (**C**) PCA of single-cell RNA-seq on neurons from fetal quiescent cells (16- to 18-week post-conception; red), fetal replicating cells (16- to 18-week post-conception; green), adult temporal cortex (21–63 years old; blue) and iPSC-derived cortical neurons (black).

In order to assess how well the transcriptomes of our iPSC-derived cortical neurons reflected those of single neurons from human fetal or adult neurons, we generated single-cell RNA-seq data from sixteen 72-day-old iPSC-derived cortical neurons. Single-cell gene expression estimates from single-cell whole transcriptomics correlated well with our previous single-cell multiplex RT-qPCR data (*r* = 0.84, *P* < 10^−16^; Supplementary Material, Fig. S4). Single iPSC-derived neurons clustered closely with fetal but not adult cortical neurons and expressed high levels of genes specific for post-mitotic fetal neurons (Fig. 3A–C; Supplementary Material, Fig. S5) (20,21).

**Figure 4.**
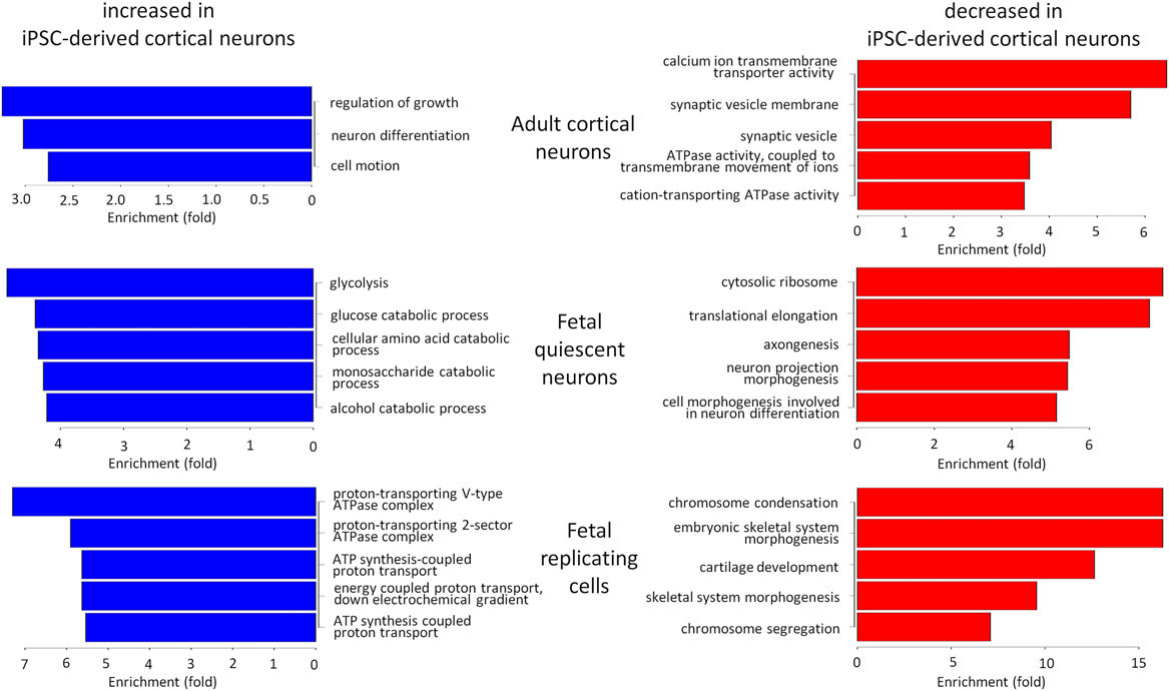
Gene ontology enrichment within differentially expressed gene lists. Bar plots of enrichment of gene ontology terms within lists of significantly differentially expressed genes relative to 72-day-old iPSC-derived cortical neurons (*n* = 16). Blue bars show enrichment for genes with higher expression in iPSC-derived cortical neurons than primary cells, and red bars show enrichment for genes with higher expression in primary cells than iPSC-derived cortical neurons. All enrichments are significant at FDR < 0.05 and are ordered by the magnitude of enrichment.

As clearly shown by clustering analysis, iPSC-derived cortical neurons show a striking resemblance to primary fetal cortical neurons at the single-cell level. iPSC-derived cortical neurons clustered more closely with primary fetal neurons, which were cultured for 3 weeks prior to single-cell transcriptomic analysis, than with freshly harvested fetal neurons (Fig. 3A). This suggests that there is a considerable effect of *in vitro* maintenance on cellular transcriptomes. However, future protocols may refine this similarity, and so we analysed our single-cell RNA-seq in order to identify functional pathways that showed significant differences between primary neurons and iPSC-derived cortical neurons (Fig. 4). Genes identified as significantly more highly or lowly expressed in primary brain cells than in iPSC-derived cortical neurons by Monocle were submitted to DAVID for gene ontology analysis (22). Pathways more active in iPSC-derived neurons than fetal neurons included glycolysis and amino acid catabolism, whereas those more active in primary fetal cortical neurons than in iPSC-derived neurons included ribosomal and neuronal morphogenic pathways (Supplementary Material, Table S2). As expected, there was considerably less expression of synaptic or ion channel-related pathways in iPSC-derived neurons than in adult cortical neurons, indicating that these cells remain electrophysiologically immature compared with mature brain tissue.

### Cortical layer identity

**Figure 5.**
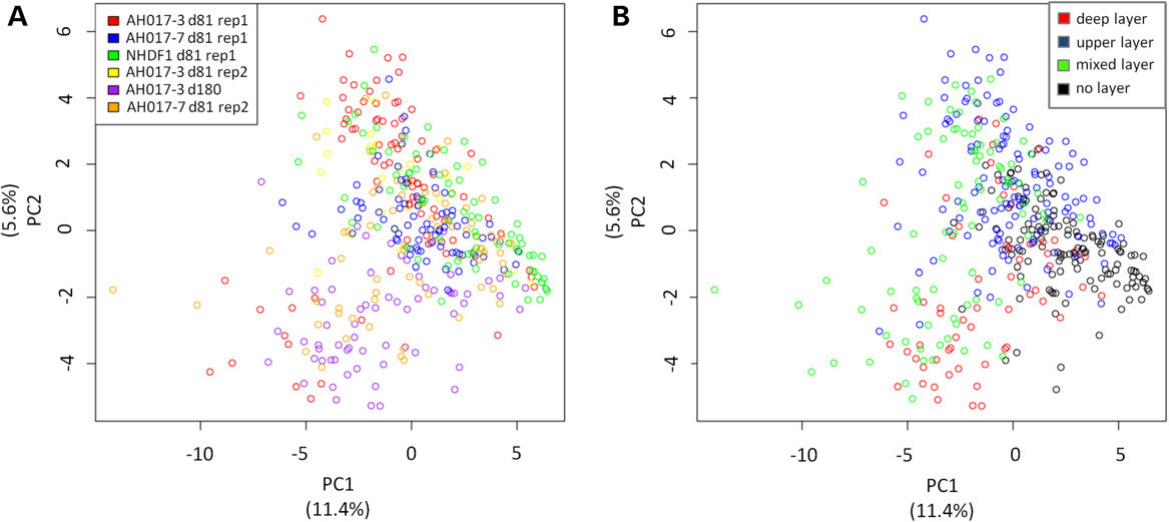
Principal component analysis. (**A**) Clustering of cells by experiment on single-cell RT-qPCR data. The percentage of total variance accounted for by each principal component is shown beneath each axis. (**B**) Clustering of cells by cortical layer identity. The percentage of total variance accounted for by each principal component is shown beneath each axis.

Principal component analysis (PCA) showed that most experiments clustered closely together (Fig. 5A), suggesting that this protocol is robust and reproducible. Prior work has established that there is continuous neurogenesis in these *in vitro* cultures with deep layer neurons emerging early and upper layer neurons appearing late (2,4). We therefore examined whether cells clustered by cortical layer identity. Cortical layer markers implicated in layer specification as a result of bulk transcriptomic approaches in human brain were expressed only patchily in iPSC-derived cortical neurons and so were not considered as likely markers of layer identity in these cultures (Supplementary Material, Table S3) (17). There was no obvious clustering by cortical layer identity as assessed by canonical layer markers [Fig. 5B; deep layer markers: *BCL11B* (also known as CTIP2) and *TBR1*; upper layer markers: *CUX1*, *POU3F2* (also known as BRN2) and *SATB2*]. There is a suggestion of separation between cells of deep and upper layer identity on PCA, but this fell below statistical significance after correction for multiple-hypothesis testing (*P* = 0.07).

**Figure 6.**
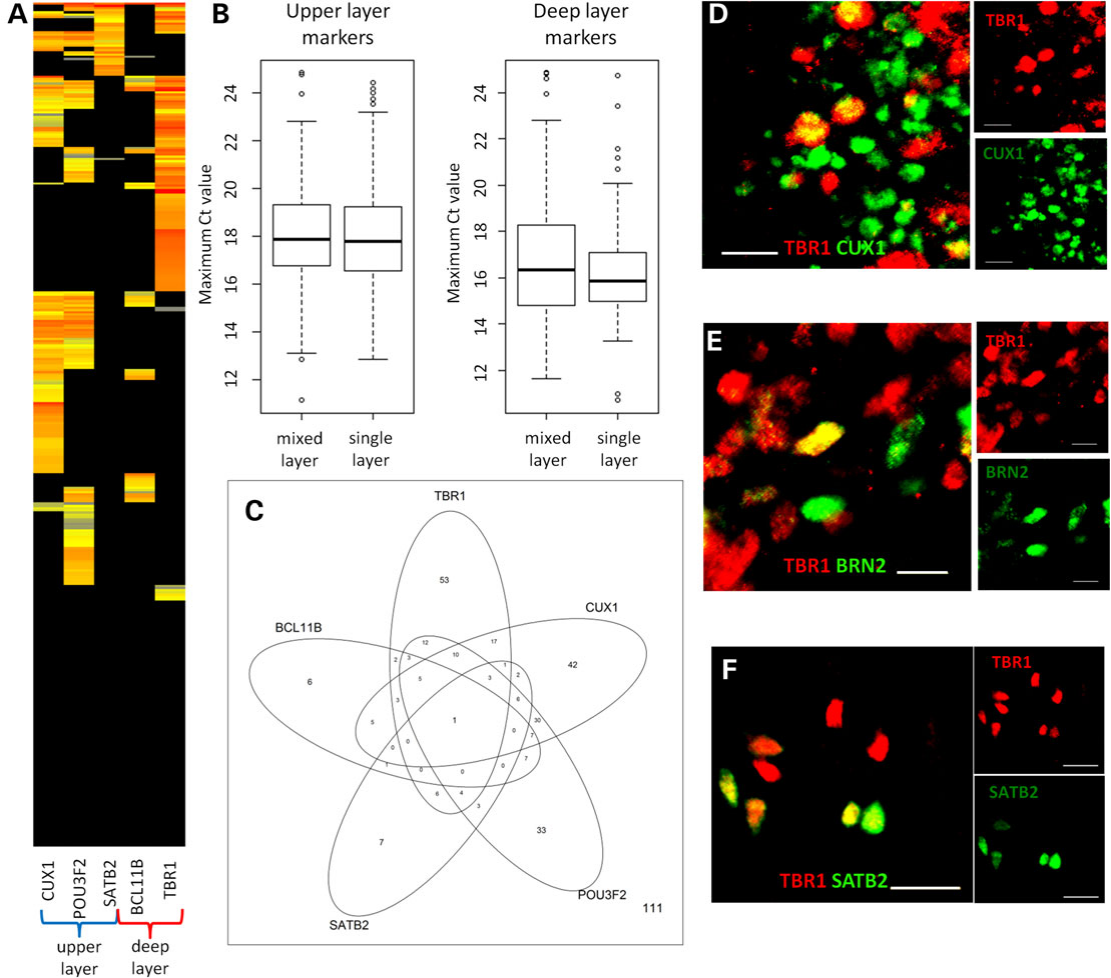
Cells with mixed cortical layer identity. (**A**) Heat map of single-cell gene expression data in neurons (*n* = 380) for deep and upper layer markers. Neurons (rows) are clustered by Euclidean distance. (**B**) Box plots showing the magnitude of cortical layer marker expression by RT-qPCR in cells expressing both deep and upper layer markers or those with exclusively deep or upper layer identity. (**C**) The number of cells expressing each cortical layer marker by RT-qPCR. (**D–F**) Immunofluorescent images of cells co-expressing the deep layer marker TBR1 (red) and the upper layer markers CUX1 (**D**), BRN2 (**E**) or SATB2 (**F**) (green). Immunofluorescent images were from cortical neurons aged >81 days derived from AH017-3 (**C** and **D**) or NHDF1 (**E**) iPSCs. Scale bars represent 20 μm.

We sought to investigate further this apparent lack of clustering. Of the 380 iPSC-derived cortical neurons, 184 (48.4%) could be unambiguously assigned to either deep or upper layer identity on the basis of canonical layer markers with our single-cell RT-qPCR dataset. A total of 111 (29.2%) neurons had no detectable expression of any of the layer marker genes. And, 85 (22.4%) neurons expressed mixed cortical layer markers (Fig. 6A). Potentially any clustering could have been obscured by cells with no or mixed layer identity. We considered whether cells of mixed layer identity could be those with low expression of one set of canonical layer markers in combination with otherwise well-established layer identity. However, the level at which layer markers were expressed did not appear to differ between neurons of single or mixed layer identity (Fig. 6B; *P* > 0.05 for all; correlation between maximum deep and upper layer gene expression in cells of mixed layer identity: *r*^2^ = 0.001, *P* = 0.76). Almost all combinations of canonical layer markers were expressed in cells (Fig. 6C).

Immunofluorescence microscopy demonstrated that this layer marker co-expression translated into protein co-expression (Fig. 6D–F). We quantified the degree of layer co-expression for TBR1 and upper layer markers by immunofluorescence co-localization using well-established antibodies (Supplementary Material, Table S4) (2).

Interestingly, although some combinations of markers showed the patterns of co-expression in our multiplex RT-qPCR dataset that would be expected from laminar identity (*CUX1* versus *POU3F2*: *r* = 0.27, *q* < 0.0002; *POU3F2* versus *SATB2*: 0.14, *q* = 0.03), the canonical deep layer marker *BCL11B* was correlated with the upper layer markers *CUX1* (*r* = 0.14, *q* = 0.02) and *POU3F2* (*r* = 0.23, *q* = 0.001) (Supplementary Material, Table S5).

We then re-examined RNA-seq data from primary human cells and made the surprising observation that mixed layer identity is apparent in a proportion of single-cell transcriptomes from fetal and adult brains (quiescent fetal cells: 66.4%; replicating fetal cells: 32.0%; adult neurons: 33.3%; Supplementary Material, Fig. S6). There was no difference between the level of expression of canonical layer markers between cells of single layer and mixed layer identity (*P* > 0.05).

## Discussion

We have confirmed that iPSC-derived neurons derived with a widely used protocol will generate neocortical cells principally expressing markers of neuronal excitatory glutamatergic phenotype at a single-cell level. These cells accurately recapitulated the single-cell transcriptomic signature of human fetal cortical neurons, and expressed many genes associated with neuronal and synaptic function. Widely used cortical layer markers were able to assign the majority of cells unambiguously to deep or upper layer identity, but a considerable minority of cells co-expressed deep and upper layer markers.

Previous studies have compared bulk transcriptomic profiles of iPSC-derived cortical neurons to primary brain tissue (23,24). When iPSC-derived cortical neurons were compared with donor-matched post-mortem brain, the similarity between iPSC-derived and primary cells increased with extended time in culture (23). Another group showed that iPSC-derived neurons closely resembled fetal brain and that the equivalent ‘age’ of the cultured cells was increased by changing from a two-dimensional (2D) to three-dimensional (3D) *in vitro* system (24). However, any comparison between cultured cells and primary tissue will be hampered by alterations in the ratios between different cell types. For example, the size of the glial population in fetal brain will change markedly overtime, and so the eventual production of glia in iPSC-derived cultures could well increase the apparent similarity of cultured cells to more mature primary tissue without any change in the underlying neuronal transcriptome (4). Single-cell transcriptomics offers a method of assessing the similarity of iPSC-derived cells to primary tissue with the ability to remove the confounding introduced by cellular heterogeneity. Our study shows that even at the level of individual cells, there is marked similarity to human fetal neurons (20,21).

There are clearly still transcriptomic differences between iPSC-derived cortical neurons and primary fetal cells, as highlighted by our differential gene analysis. iPSC-derived cortical neurons show higher expression of genes related to glycolysis and catabolism of amino acids than is the case for fetal neurons *in vivo*, which may be a result of the abnormality of *in vitro* conditions. Similarly, the lack of structured cell migratory pathways *in vitro* was clear from the transcriptomic signature of iPSC-derived cells, with markedly lower expression of *DCX*, *ROBO2* and other genes involved in neuronal motility. Relatively simple 2D adherent monolayer neuronal cultures are more amenable for high-throughput screens, but potentially more organized, 3D models may improve matters. However, without a morphologically defined ventriculo-pial gradient *in vitro*, there may still be deficits in differentiation and migration (24). The fact that iPSC-derived cortical neurons cluster distinctly from adult cortical neurons may result in issues when attempting to model neurodegenerative disorders using these systems. This means that care should be taken when making molecular inferences regarding adult-onset neurodegenerative diseases from studies in iPSC-derived systems (25). This is supported by the lack of detectable expression of many adult markers of cortical neurons and suggests that iPSC-derived cortical neurons are relatively immature compared with primary brain neurons. Regional specification of the iPSC-derived cortical neurons is also difficult to assess, as some of the key markers of neocortical regions are transcription factors that are expressed at relatively low level and may therefore be missed by a single-cell transcriptomic approach. Our gene ontology enrichment analysis suggested that the expression of some key genes involved in neuronal development was lower in iPSC-derived cortical neurons than in primary adult or fetal neurons (e.g. *CDK5R1*, *LPPR4*, *PTPRZ1*, *BCL11B*, *MAP1B*, *ROBO2*, *NRXN1*, *DCX*, *GAS7*, *RPS27A*). Hopefully attention to these pathways may help guide improvements to existing differentiation protocols. Despite these limitations, stem cell-derived models of corticogenesis are still likely to represent the best *in vitro* human model amenable to molecular manipulation of cells for the foreseeable future (26).

Many of the canonical layer markers used to infer cortical layer identity in iPSC-derived cortical neurons have been informed primarily by rodent models of corticogenesis (12). It is perhaps therefore surprising that they were able to assign the majority of single-cell transcriptomes generated from iPSC-derived neurons to either deep or upper layer identity. However, there was clearly a subpopulation of neurons that had mixed layer identity.

Unexpectedly, this appeared also to be the case when we examined layer identity in available single-cell RNA-seq data from 21-week post-conception fetal and adult cortical neurons (20,21). This may represent neurons with truly mixed deep and upper layer identities, or alternatively, the cortical layer markers widely used to assess layer identity do not function well in a minority of cells. For the primary neuron data, it is also possible that there was spill-over between C1 capture sites in the primary brain single-cell RNA-seq experiments, but we feel this is unlikely to be the case because two empty C1 capture sites had no detectable expression of any of the presumptive layer markers. This would also not explain the co-occurrence of deep and upper layer markers in our multiplex RT-qPCR dataset, which was obtained by FACS.

Longitudinal bulk transcriptomic data obtained from cortical neurons-derived from human embryonic stem cells have shown that genes regarded as upper layer markers actually appear to peak early in differentiation (27). Whether or not this gene expression signature is driven by neurons is impossible to ascertain from bulk transcriptomic approaches. Our single-cell data and analysis of published data suggest that some single cortical neurons can co-express multiple layer markers. When we extended the duration that the cells remained in culture out to 180 days (∼25 weeks), we detected a high proportion of mixed layer identity, suggesting that this is not simply a transitory state in neuronal differentiation, at least *in vitro*. In fact, we found that the proportion of different subpopulations of cells detected did not vary significantly between 81 and 180 days. We feel that the few non-neuronal cells detected in our cultures are likely to be neural progenitors.

Many fetal layer markers show reciprocal regulation, and in rodent models of corticogenesis, mixed layer identity may indicate aberrant neural development (28,29). Later stages of fetal corticogenesis demonstrate defined distributions of layer-specific markers as detected by immunohistochemistry (13). Whether this co-expression is detectable at the protein level in human cortex is unclear and should be examined in future studies. However, we observed the translation of layer marker co-expression into protein in iPSC-derived cortical neurons. This may represent neurons with truly mixed deep and upper layer identities, or alternatively, the cortical layer markers widely used in iPSC-derived cortical neuron cultures to assess layer identity do not function well in a minority of cells.

Mixed cortical layer identity is a potential limitation to consider when modelling neurological diseases, particularly those in which layer identity is important, such as Alzheimer's disease and many neurodevelopmental conditions, since, unlike *in vivo*, spatial distribution cannot be used as a proxy for cortical layer identity (16,28,30,31). Our findings suggest that layer identity may be better established by combining single-cell transcriptomics with functional read-outs such as electrophysiology than by relying on the expression of single transcription factors.

iPSC-derived cortical neurons appear to recapitulate accurately the transcriptome of human fetal corticogenesis but are distinct from adult neurons. Canonical layer markers traditionally used to assign cells to cortical layers are capable of assigning the majority of cells unambiguously to either deep or upper layer identity. However, a subpopulation of cells shows mixed layer identity both at the level of RNA and protein expression. A degree of mixed layer identity also appears to be present in primary cortical neurons. Overall, our findings suggest that protocols to derive cortical neurons from iPSCs are a good model of fetal corticogenesis and produce cells that accurately mimic *in vivo* fetal cortical neurons.

## Materials and Methods

### Ethics statement

The human hiPS cell lines derived for this study were derived from human skin biopsy fibroblasts, following signed informed consent, with approval from a research ethics committee: National Health Service, Health Research Authority, NRES Committee South Central—Berkshire, UK, who specifically approved this part of the study—REC 10/H0505/71.

### Derivation of iPSCs

iPSC lines were derived from skin biopsy fibroblasts in the James Martin Stem Cell Facility, University of Oxford, and all cultured under standardized protocols to minimize any potential variation attributable to laboratory differences and/or handling. Fibroblasts and derived iPSC lines tested negative for mycoplasma using MycoAlert (Lonza). iPSC-NHDF1 (44-year-old female, reprogrammed with Yamanaka retroviruses *SOX2*, *KLF4*, *OCT3/4*, *c-MYC* and *NANOG*) has been described previously (32). iPSC-AH017-3 and iPSC-AH017-7 (67-year-old female) are described for the first time here. They were derived using the SeVdp(KOSM)302L Sendai virus system, containing genes for *KLF4*, *OCT3/4*, *SOX2* and *c-MYC* expressed from a single transcript, packaged into a single Sendai virus, ensuring consistency of gene dosage ratio (33,34). The system also contains a target for mir302; mir302 is expressed in pluripotent cells, but not in the originating fibroblasts, ensuring complete removal of exogenous genetic material within a few passages.

Sendai-transduced fibroblasts were transferred onto mitomycin C-inactivated mouse embryonic feeder cells [MEFs; outbred Swiss mice established and maintained at the Department of Pathology, Oxford (35,36)] on 0.1% gelatine-coated plates on Day 2 and cultured in iPS medium consisting of knock-out DMEM (Invitrogen), 10% knock-out-Serum Replacement (Invitrogen), 2 mm Glutamax-I (Gibco), 100 U/mL penicillin (Invitrogen), 100 µg/mL streptomycin (Invitrogen), 1% non-essential amino acids (Invitrogen), 0.055 mm β-mercaptoethanol (R&D) and 10 ng/mL bFGF (R&D). Medium was replaced (50%) daily, substituting with MEF-conditioned medium from Day 10 onwards. Colonies displaying iPSC morphology were picked on ∼Days 21–28 and manually dissected onto fresh feeders every 5–7 days until stabilized, then adapted to feeder-free conditions onto Matrigel-coated plates (BD Matrigel hESC-qualified Matrix) in mTeSR™1 (StemCell Technologies, 100% change daily). Bulk passaging was by 0.5 mm ethylenediaminetetraacetic acid (EDTA) to make large-scale, quality-controlled, cryopreserved stocks (genomic RNA and RNA extraction for results shown is passage 22 for iPSC-AH017-3 and passage 17 for iPSC-AH017-7) (37). The number of feeder-free passages was kept to a minimum, to ensure that differentiation experiments were set up within a consistent and very narrow window of passage numbers.

### Assessment of genome integrity, and tracking

Genomic DNA was extracted using Qiagen Blood and Tissue Kit. Genome integrity was assessed by an Illumina Human CytoSNP-12v2.1 beadchip array (∼300 000 markers), analysed using KaryoStudio software (Illumina) and SNP deviations in the iPSC lines compared with the original pool of fibroblasts. This also enabled confirmation of the identity of the iPSC to the original fibroblasts. SNP datasets have been deposited in GEO under the accession number GSE69287 (Superseries GSE69302).

### Sendai clearance assay

Clearance of Sendai virus from the reprogrammed cells was confirmed by RT-qPCR. RNA was extracted using an RNeasy kit (Qiagen) from iPSC, from fibroblasts (negative control) and from fibroblasts infected with the Sendai reprogramming virus 5 days previously (positive control). Reverse transcription was carried out using a RetroScript kit (Ambion; 2 μg template RNA, 20 μl reaction volume), then 2 μl of 1:10 cDNA product in a 25 μl RT-qPCR reaction (Applied Biosystems StepOne Plus Real Time PCR machine, StepOne software), using Applied Biosystems 2xSYBR green PCR mix + ROX, 60°C anneal and Sendai-specific primers (5′ AGACCCTAAGAGGACGAAGACAGA 3′ and 5′ ACTCCCATGGCGTAACTCCATAG 3′). Target gene transcript levels were compared with actin B control (actin B primers, Eurogentec), and subsequently to the positive control (38).

### Pluritest

RNA was extracted from iPSC using an RNeasy kit (Qiagen) for Illumina HT12v4 transcriptome array analysis. Image data files were uploaded to www.pluritest.org and scored for pluripotency as previously described (39). Transcriptome datasets have been deposited in GEO under the accession number GSE69288 (Superseries GSE69302).

### Flow cytometry

iPSC lines were stained for expression of the pluripotency marker using TRA-1-60 Alexa Fluor 488 conjugate (B119983, Biolegend; with IgM-488 isotype-matched Alexa Fluor 488 control). Briefly, cells were fixed with 4% paraformaldehyde in phosphate-buffered saline (PBS) and permeabilized with methanol at −20°C. A total of 0.5–1 × 10^6^ cells were washed and stained in flow cytometry buffer [PBS, human IgG (10 µg/mL Sigma), FCS (1% Hyclone) sodium azide (0.01%)], for 30 min. The cells were assayed using a FACSCalibur (Becton Dickinson)—data from 10 000 cells were collected and analysed using FlowJo software, gated on FSC-SSC dot plots, and plotted as histograms (*y*-axis normalized to mode).

### Cortical neuronal differentiation

iPSCs were differentiated into cortical neurons using the Livesey protocol with some modifications (2). In brief, iPSCs were cultured in an adherent monolayer on matrigel-coated plates. Neural induction was achieved using dual SMAD inhibition (1 µM dorsomorphin and 10 µM SB431542) in neural maintenance media (DMEM/F-12, neurobasal, N-2, B-27, 5 µg/ml insulin, 1 mm l-glutamine, 100 µM non-essential amino acids, 100 µM 2-mercaptoethanol, 50 units/ml penicillin and 50 mg/ml streptomycin). After the formation of a neuroepithelial sheet, cells were passaged as small clusters onto wells coated with poly-l-ornithine and laminin. The subsequent differentiating cells were allowed to form neural rosettes in the presence of b-FGF and passaged until confluent and frozen as cortical neural progenitor stocks. RT-qPCR and immunofluorescence microscopy confirmed the adoption of cortical identity. Cortical cultures were maintained in neural maintenance media with additional laminin feeding (100 µg/ml) every 10 days to avoid cell detachment. Neuronal age was reckoned from the day of neural induction. Cells were treated with 4 μM cytosine arabinoside for 72 h prior to single-cell analysis.

### Selection of genes for RT-qPCR

Candidate genes related to cortical layer or region identity and neuronal function were curated from multiple sources (2,12,13,17–19). *GAPDH* and *ACTB* were included as housekeeping genes. Several glial genes were included to identify glia. Negative control genes normally expressed solely in the kidney and liver were also included. Primer sequences are shown in Supplementary Material, Table S3.

### Bulk RNA extraction and reverse transcription

RNA was extracted from entire wells by using the RNeasy Micro Kit (Qiagen) and reverse transcribed using SuperScript III (Invitrogen).

### Single-cell RT-qPCR

Single-cell suspensions were generated using accutase dissociation followed by single-cell filtration of iPSC-derived cortical neurons aged >81 days. The success of the suspension was manually confirmed on a haemocytometer. The single-cell suspension was sorted into a 96-well PCR plate containing a lysis mix [VILO reaction mix (Invitrogen), SUPERase-In (Ambion) and 10% NP40 (Fisher Scientific)] using a cell sorter (Becton Dickinson FACSAria II SORP or FACSAria Fusion). Sorting gates were set to include only live (DAPI negative) single cells. Stream alignment and sort efficiency was checked using Accudrop beads (Becton Dickinson). The PCR plate was vortexed and centrifuged before performing denaturation at 65°C for 90 s. RT mix [SuperScript enzyme mix (Invitrogen) and T4 Gene 32 Protein (New England Biolabs)] was added to this, and then this mix was reverse transcribed (25°C for 5 min, 50°C for 30 min, 55°C for 25 min, 60°C for 5 min and 70°C for 10 min). Preamplification was then performed with TaqMan PreAmp Master Mix (Invitrogen), 500 nM Deltagene assay primer mix (Fluidigm) and 0.5 M EDTA, pH 8.0 (Invitrogen) on a thermocycler (95°C for 10 min, and 20 cycles of 96°C for 5 s and 60°C for 4 min). The resultant product was treated with exonuclease I (New England Biolabs) to remove unincorporated primers (37°C for 30 min and 80°C for 15 min). Individual primers (100 µM) were combined with assay loading reagent (Fluidigm) and TE DNA suspension buffer (Invitrogen) before being pipetted onto a Biomark chip (Fluidigm). The cDNA from the 96-well PCR plate was diluted with DNA suspension buffer 1 in 5, combined with sample loading reagent (Fluidigm) and loaded onto the same Biomark chip. This was run on a Biomark analyser system (Fluidigm). The results were visualized using Real Time PCR Analysis software version 4.1.2 (Fluidigm). Melting curves were individually visualized for each reaction to ensure that only a single peak was selected for expression analysis and that temperature ranges were common to all experiments. Detection thresholds (Tm) were automatically generated using a baseline linear correction model and a quality threshold of 0.65.

### Single-cell RNA-seq

Cells were dissociated as for single-cell RT-qPCR at Day 72 of neuronal differentiation. A total of 300 000 DAPI negative cells were sorted into 200 μL of neural maintenance media. These were loaded onto a small C_1_ chip according to the Smarter-seq protocol detailed by Fluidigm (20,40). Cells were co-stained with Hoechst and propidium iodide. Capture chambers were imaged on an Opera Imaging System. We continued with lysis, reverse transcription, amplification and library prep in accordance with the Fluidigm Smarter-seq protocol. cDNA from 16 harvest chambers containing cells which were positive for Hoechst and negative for propidium iodide were sequenced using a rapid sequencing run (100 base paired-end reads) on an Illumina HiSeq system to a mean depth of ∼5 million reads per cell (archived as accession number GSE69790). Additional single-cell RNA-seq data were downloaded from Pollen *et al.* (Accession Number SRP041736) and Darmanis *et al.* (Accession Number GSE67835) (20,21).

### Immunofluorescence microscopy

Cells were fixed with 4% paraformaldehyde and permeabilized with 0.3% Triton-X100. These were then incubated with blocking buffer before applying the primary antibody. After washing with PBS, fluorochrome-conjugated secondary antibodies were applied. This was further washed and mounted using Dako fluorescence mounting medium on a glass slide for confocal microscopy. Antibodies were used as detailed in Shi *et al.* (2). The specificity of these antibodies was tested by staining undifferentiated iPSC and demonstrating no detectable signal withantibodies against NANOG as a positive control. Analysis of layer marker co-expression was performed by Cytospin (Thermo Scientific) preparation of dissociated iPSC-derived cortical neurons as described in (2) followed by the counting of five fields of view stained by DAPI and for various combinations of layer markers. Results are presented as mean proportions ± standard deviation.

### Electrophysiology

A coverslip containing iPSC-derived cortical neurons was placed in a recording chamber mounted onto the stage of an upright microscope, and the somata of cortical neurons were targeted for recording using IR-DIC optics. Cells were continuously superfused with oxygenated aCSF (95% O_2_/5% CO_2_) containing 130 mm NaCl, 25 mm NaHCO_3_, 2.5 mm KCl, 1.25 mm NaH_2_PO_4_, 2 mm CaCl_2_, 2 mm MgCl_2_ and 10 mm glucose. Patch-clamp electrodes (4–7 MΩ) were filled with an intracellular solution containing 120 mm K-gluconate, 10 mm KCl, 10 mm HEPES, 4 mm MgATP, 0.3 mm NaGTP and 10 mm Na-phosphocreatine. Recordings were obtained using a Multiclamp 700B amplifier and digitized at 10–20 kHz using Digidata 1550 acquisition board.

### Data analysis

Single-cell RT-qPCR data were analysed in R. In accordance with guidelines on single-cell RT-qPCR analysis, Ct values were analysed without adjusting for housekeeping gene expression levels (9). Genes with detectable expression were defined as those with a Ct value of <30. A well was defined as containing a cell that had been successfully reverse transcribed if there were detectable levels of *GAPDH* (Ct < 30). We tested primer efficacy with single-cell cDNA by conducting a dilution series and found good linear amplification for detectable genes (*r*^2^ = 0.98 ± 0.03; Supplementary Material, Fig. S7A). Genes were excluded from downstream analysis if these showed poor inter-chip reliability (the same single-cell cDNA analysed on different chips). There was excellent correlation of gene expression between technical replicates (median *r*^2^ = 0.96 ± 0.05; Supplementary Material, Fig. S7B). Ct values were adjusted between batches by the difference in mean *GAPDH* and *ACTB* expression relative to the first replicate of AH017-3. Cells were classified as neurons if there was detectable expression of *MAP2*, *NCAM1* or *TUBB3*. Cortical layer identity was established through the expression of candidate layer markers: deep layer markers: *BCLB11* and *TBR1*; upper layer markers: *CUX1*, *POU3F2* and *SATB2*. Internal controls were also used to estimate the magnitude of technical variation between Biomark analysis runs. The significance of PCA clustering was calculated by applying a Wilcoxon test to the distance between individual centroid locations for each group of points to be analysed compared with the distance to the centroid for the pooled points of both groups. Co-expression was analysed by calculating correlation coefficients on the original dataset for pairs of cortical layer markers and then randomly permuting these Ct values 10 000 times. An empirical two-tailed *P*-value was estimated from this distribution and corrected for multiple-hypothesis testing using the Benjamini–Hochberg method.

RNA-seq reads were processed using Trimmomatic with the arguments ‘LEADING:3 TRAILING:3 SLIDINGWINDOW:4:15 MINLEN:50’ (41). Trimmed reads were aligned against the hg19 UCSC genome assembly using Tophat2 (version 2.0.13, arguments ‘-p 8 -G < genome bt2 index >−1 < forward reads >−2 < backward reads>’) (42). Overall alignment rates for iPSC-derived cortical neurons were ∼67% with ∼4000 detectable genes per cell. Fragments were assigned to UCSC known genes with Rsubread (version 1.16.1, arguments ‘isGTFAnnotationFile = TRUE, GTF.featureType=‘exon’, GTF.attrType=‘gene_name’, useMetaFeatures = TRUE, allowMultiOverlap = FALSE, isPairedEnd = TRUE, requireBothEndsMapped=thinsp;TRUE, checkFragLength = FALSE, nthreads = 8, strandSpecific = 0, minMQS = 0, countMultiMappingReads = FALSE, countChimericFragments = FALSE) (43). Counts were adjusted for library sizes using DESeq (version 1.20.0, estimateSizeFactorsForMatrix) (44). Cells were removed during QC if the number of expressed genes was lower that the boxplot lower limit (i.e. an outlier), if the proportion of fragments aligning to mitochondrial genes was high (>0.15 as suggested in a previous study (45)) or if the cell was an outlier on initial PCA (single cells of the same type were analysed by PCA and were excluded if >5 times the median absolute deviation from the centroid). A gene was considered detectable if at least one fragment with both paired reads uniquely mapped was assigned to it. PCA was illustrated for the top 300 genes explaining the first two principal components as well as for the whole transcriptome. Cells types of single cells from primary tissue datasets were provided by the respective study authors (20,21). Differential gene analysis of single-cell data was conducted using Monocle with an FDR threshold of 0.05 (46). We only considered genes differentially expressed if the gene was detectable in >10% of cells at a mean expression level FPKM > 1 in at least one type of cell where the direction of change in the proportion of cells expressing the gene and the magnitude of mean expression were the same. Gene ontology analysis was conducted in DAVID using all detectable genes as the background (22).

## Supplementary Material

Supplementary Material is available at *HMG* online.

## Authors’ Contributions

A.E.H.: conception and design, financial support, provision of study material or patients, collection and/or assembly of data, data analysis and interpretation, manuscript writing, final approval of manuscript.

S.C.: provision of study material or patients, collection and/or assembly of data, final approval of manuscript.

T.L.: collection and/or assembly of data, final approval of manuscript.

E.W.: provision of study material or patients, final approval of manuscript.

J.V.: provision of study material or patients, final approval of manuscript.

A.G.: collection and/or assembly of data, final approval of manuscript.

K.A.: collection and/or assembly of data, final approval of manuscript.

P.S.: collection and/or assembly of data, final approval of manuscript.

M.N.: provision of study material or patients, final approval of manuscript.

R.B.: collection and/or assembly of data, financial support, final approval of manuscript.

S.C.: provision of study material or patients, collection and/or assembly of data, final approval of manuscript.

S.N.: provision of study material or patients, final approval of manuscript.

C.A.: provision of study material or patients, final approval of manuscript.

C.P.P.: data analysis and interpretation, financial support, manuscript writing, final approval of manuscript.

M.Z.C.: conception and design, financial support, provision of study material or patients, data analysis and interpretation, manuscript writing, final approval of manuscript.

## Funding

This project was supported by StemBANCC funding from the Innovative Medicines Initiative Joint Undertaking under Grant Agreement Number 115439, resources of which are composed of financial contribution from the European Union's Seventh Framework Programme (FP7/2007e2013) and EFPIA companies in kind contribution. A.E.H. was funded by a Wellcome Trust Research Training Fellowship (100643/Z/12/Z). The Oxford Martin School (LC0910-004) and the Wellcome Trust (WTISSF121302) provide core support to the James Martin Stem Cell Facility within the Sir William Dunn School of Pathology. Samples and associated clinical data were supplied by the Oxford Parkinson's Disease Centre (OPDC) study, funded by the Monument Trust Discovery Award from Parkinson's UK, a charity registered in England and Wales (2581970) and in Scotland (SC037554), with the support of the National Institute for Health Research (NIHR) Oxford Biomedical Research Centre based at Oxford University Hospitals NHS Trust and University of Oxford, and the NIHR Comprehensive Local Research Network. The High-Throughput Genomics Group at the Wellcome Trust Centre for Human Genetics (generation of Illumina genotyping and transcriptome data) is funded by Wellcome Trust Grant Reference 090532/Z/09/Z. Funding to pay the Open Access publication charges for this article was provided by the University of Oxford Wellcome Trust open access block grant.

## Supplementary Material

Supplementary Data
