# Supplementary material for: Assessing similarity to primary tissue and cortical layer identity in induced pluripotent stem cell-derived cortical neurons through single-cell transcriptomics
Source: Hum Mol Genet. 2016 Jan 5;25(5):989–1000. doi: 10.1093/hmg/ddv637 (PMC4754051; doi:10.1093/hmg/ddv637)
Supplement: Supplementary Data [file supp_25_5_989__index.html]

Assessing similarity to primary tissue and cortical layer identity in induced pluripotent stem cell-derived cortical neurons through single-cell transcriptomics — Assessing similarity to primary tissue and cortical layer identity in induced pluripotent stem cell-derived cortical neurons through single-cell transcriptomics — Supplementary Data 

# Assessing similarity to primary tissue and cortical layer identity in induced pluripotent stem cell-derived cortical neurons through single-cell transcriptomics

## Supplementary Data

Supplementary Data

- Supplementary Figures - docx file
- Supplementary Tables - docx file
