# Supplementary material for: Assessing similarity to primary tissue and cortical layer identity in induced pluripotent stem cell-derived cortical neurons through single-cell transcriptomics
Source: Hum Mol Genet. 2016 Jan 5;25(5):989–1000. doi: 10.1093/hmg/ddv637 (PMC4754051; doi:10.1093/hmg/ddv637)
Supplement: Supplementary Data [file supp_ddv637_ddv637supp_figs.docx]

**
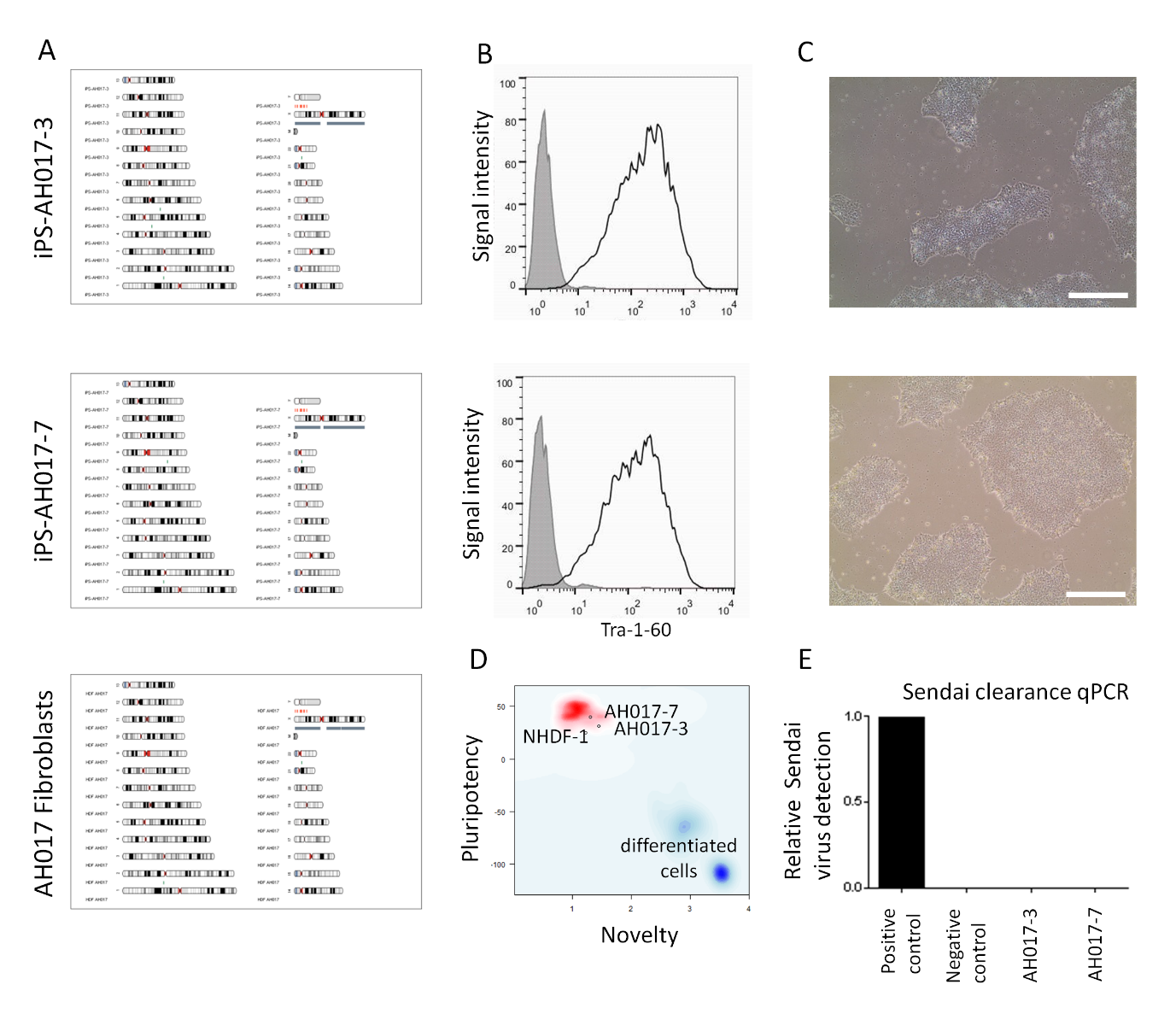
**

**Supplementary Figure 1 Characterisation data for lines iPS-AH017-3 and iPS-AH017-7.** **(A)** Karyogram showing genome integrity of iPSC (Illumina Human CytoSNP-12 beadchip, analysed using Karyostudio). Autosomal detected regions deviating from reference data are annotated with green (amplification) or orange bands (deletion); normal female X chromosome complement is indicated as grey; lower panel shows the parental fibroblasts as reference. **(B)** FACs analysis of iPSC for pluripotency marker Tra-1-60 (black line; grey filled plot, isotype control). **(C)** iPSC show hESC-like morphology (tightly packed colonies, high nucleus to cytoplasm ratio by phase microscopy; scale bar: 100µm). **(D)** PluriTest analysis of Illumina HT12v4 transcriptome array data shows iPSC clusters with pluripotent stem cells (red cloud) and not with partly- or differentiated cells (blue clouds). iPS-NHDF-1 (a previously published line (32)), also used in this study, is marked on this plot for reference - each circle represents one iPSC line; y axis Pluripotency score, x axis Novelty score. **(E)** Assessment of Sendai clearance from iPSC lines by qRT-PCR, relative to positive control.


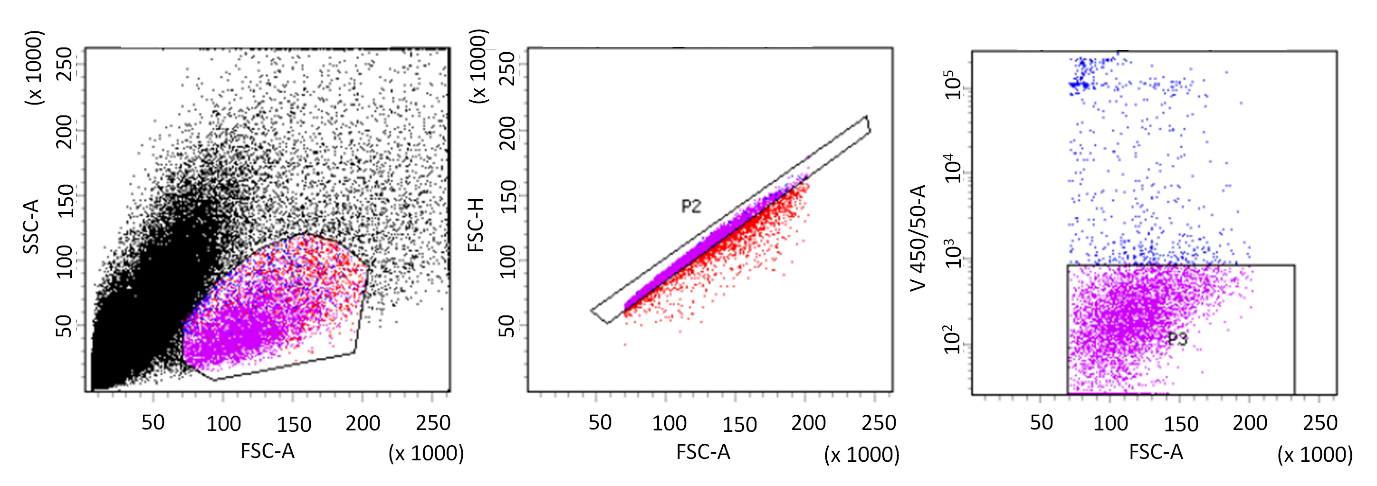


**Supplementary Figure 2 Fluorescence-activated cell sorting.** An example of a single cell FACS experiment on AH017-3 cells. 81-day old iPSC-derived cortical neurons were dissociated, passed through a 40µm filter and stained with DAPI prior to FACS analysis. The panels show selection of the cell population of interest (left), exclusion of doublets (middle) and selection of live cells (right).


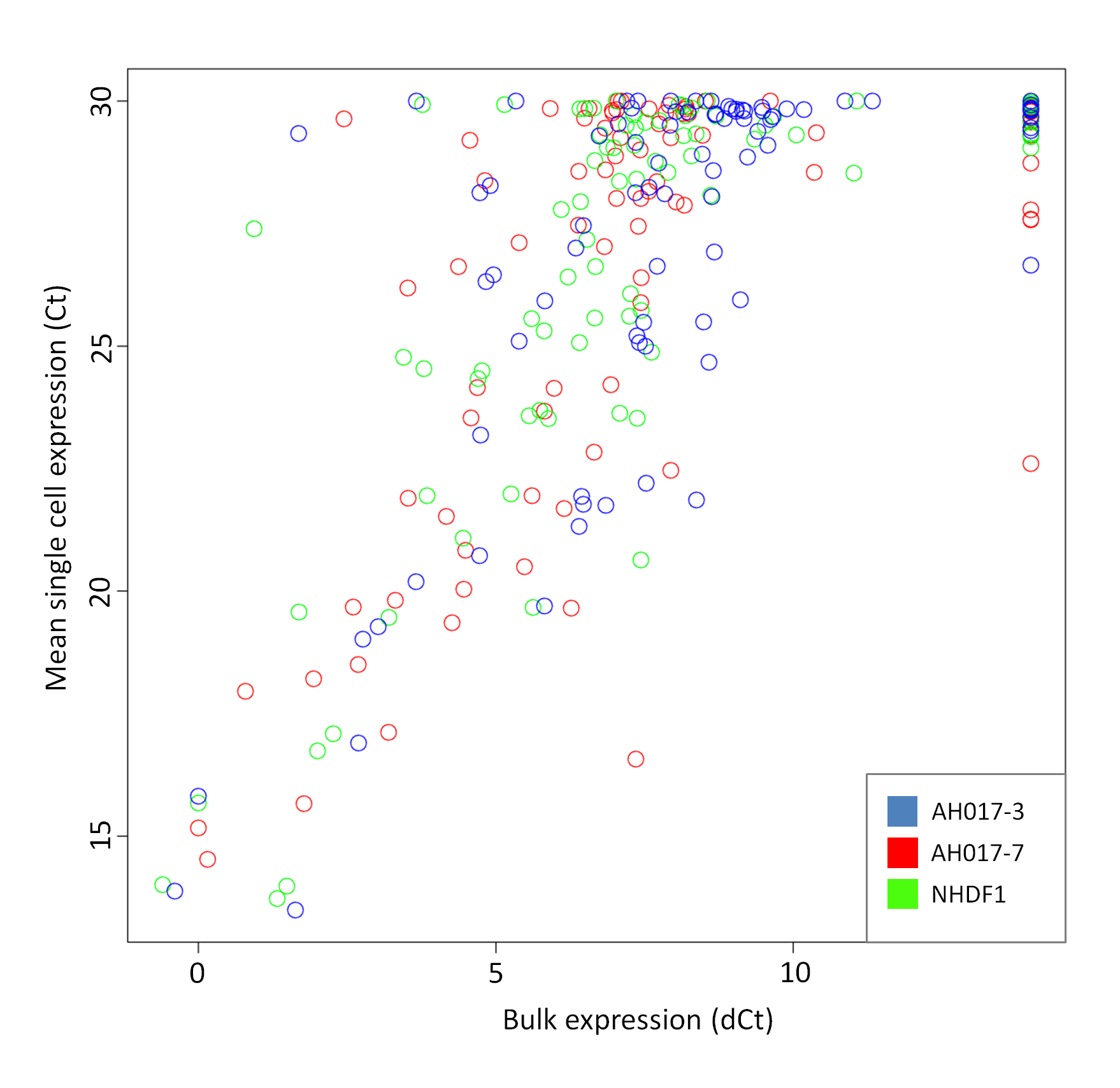


**Supplementary Figure 3 Correlation between bulk and single cell RT-qPCR**. The population dCt (normalised to GAPDH expression) from bulk samples was plotted against the mean of single cell Ct values. Cells were analysed at 81 days post-neural induction. The iPSC line from which each dataset is derived is indicated by colour.


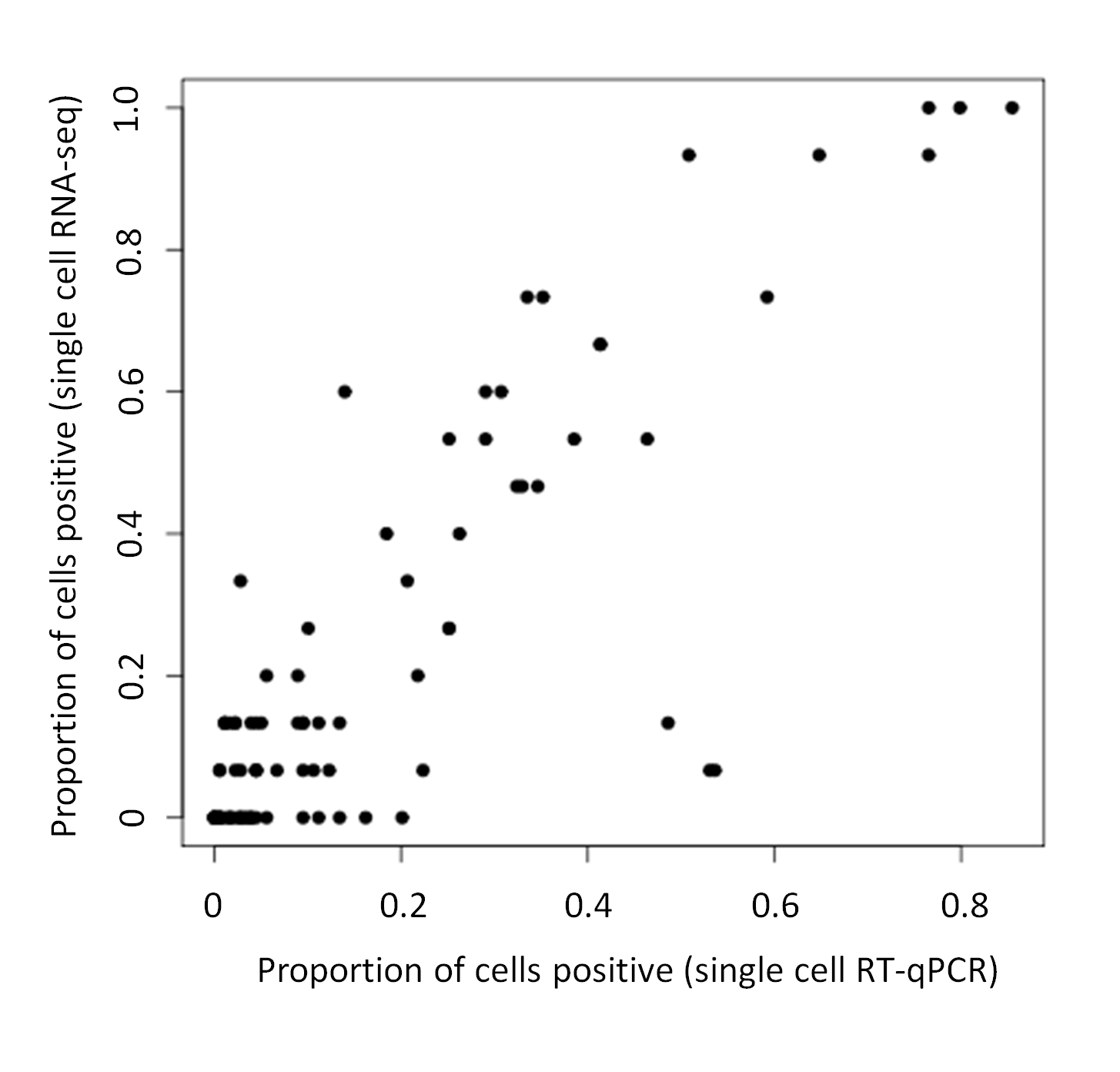


**Supplementary Figure 4 Correlation between single cell RT-qPCR and single cell RNA-seq.** Neurons were derived from AH017-7 iPSCs and are aged 81 days (RT-qPCR) or 72 days (single cell RNA-seq). The proportion of cells with detectable gene expression for RT-qPCR is shown on the x-axis and for single cell RNA-seq on the y-axis.


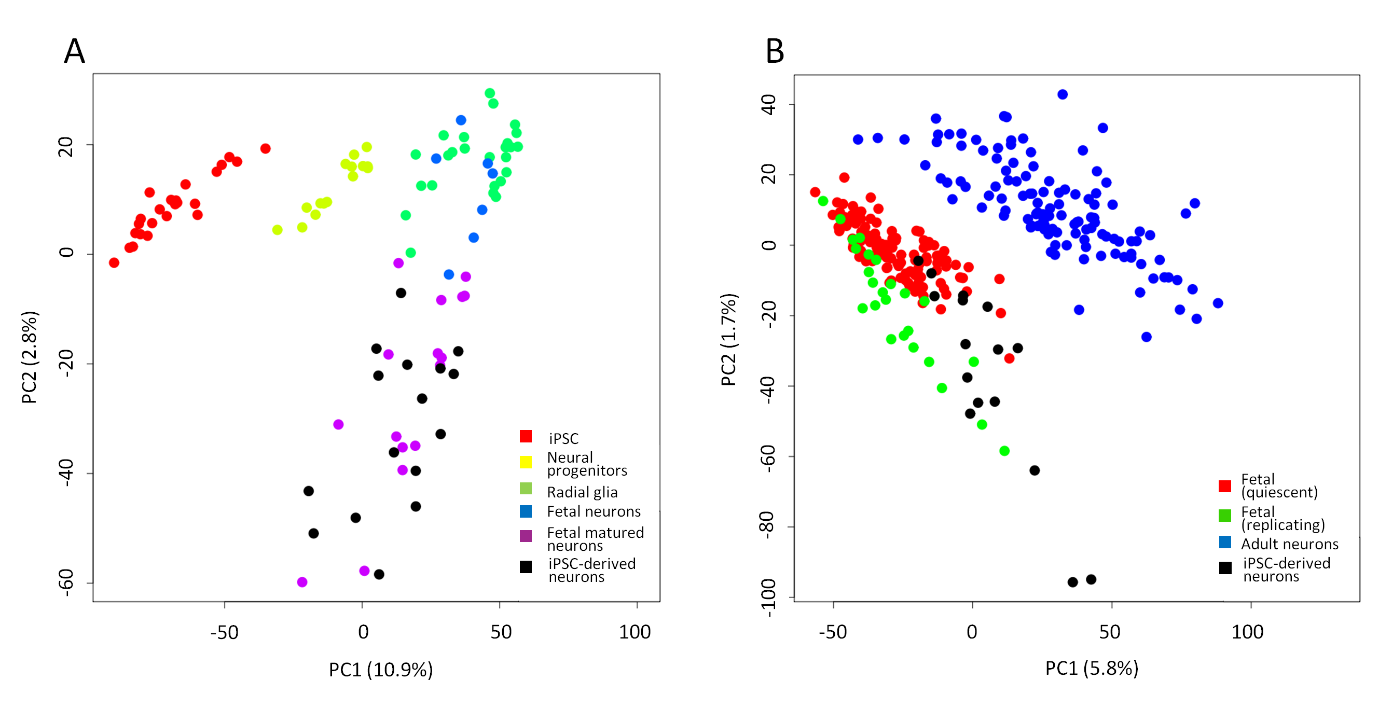


**Supplementary Figure 5 Single cell RNA-seq in iPSC-derived cortical neurons. (A)** Principal component analysis of single cell RNA-seq on iPSCs, neural progenitors, fetal radial glia, fetal newborn cortical neurons (21-weeks post-conception), fetal mature cortical neurons (21-weeks post-conception plus 3 weeks culture) and iPSC-derived cortical neurons using the whole transcriptome. **(B)** Principal component analysis of single cell RNA-seq on neurons from fetal quiescent cells (16-18-weeks post-conception), fetal replicating cells (16-18-weeks post-conception), adult temporal cortex (21 - 63 years old) and iPSC-derived cortical neurons using the whole transcriptome.


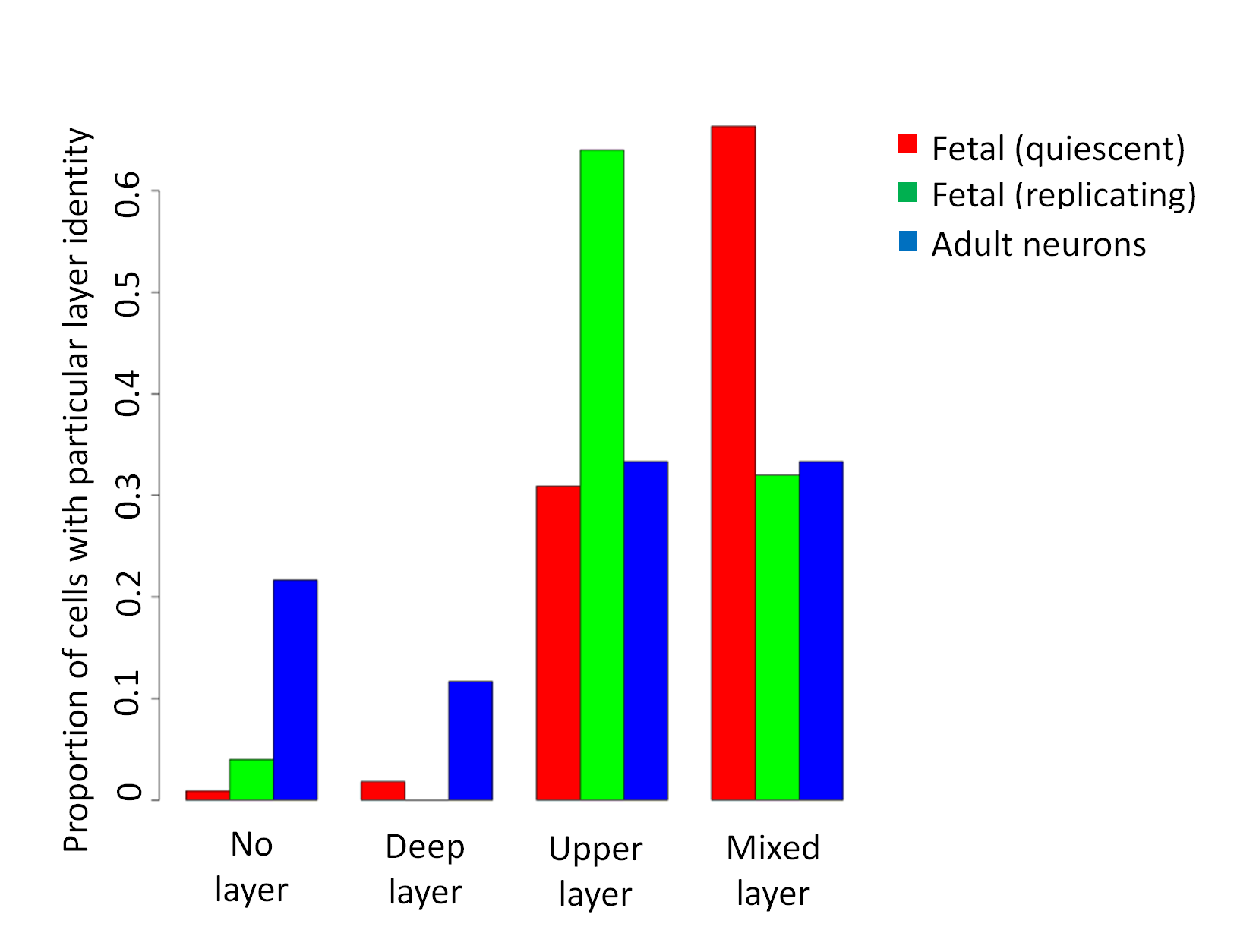


**Supplementary Figure 6 Cortical layer identity by primary brain single cell RNA-seq.** The proportion of different cell types from Darmanis *et al.* expressing different patterns of canonical fetal layer markers (21).


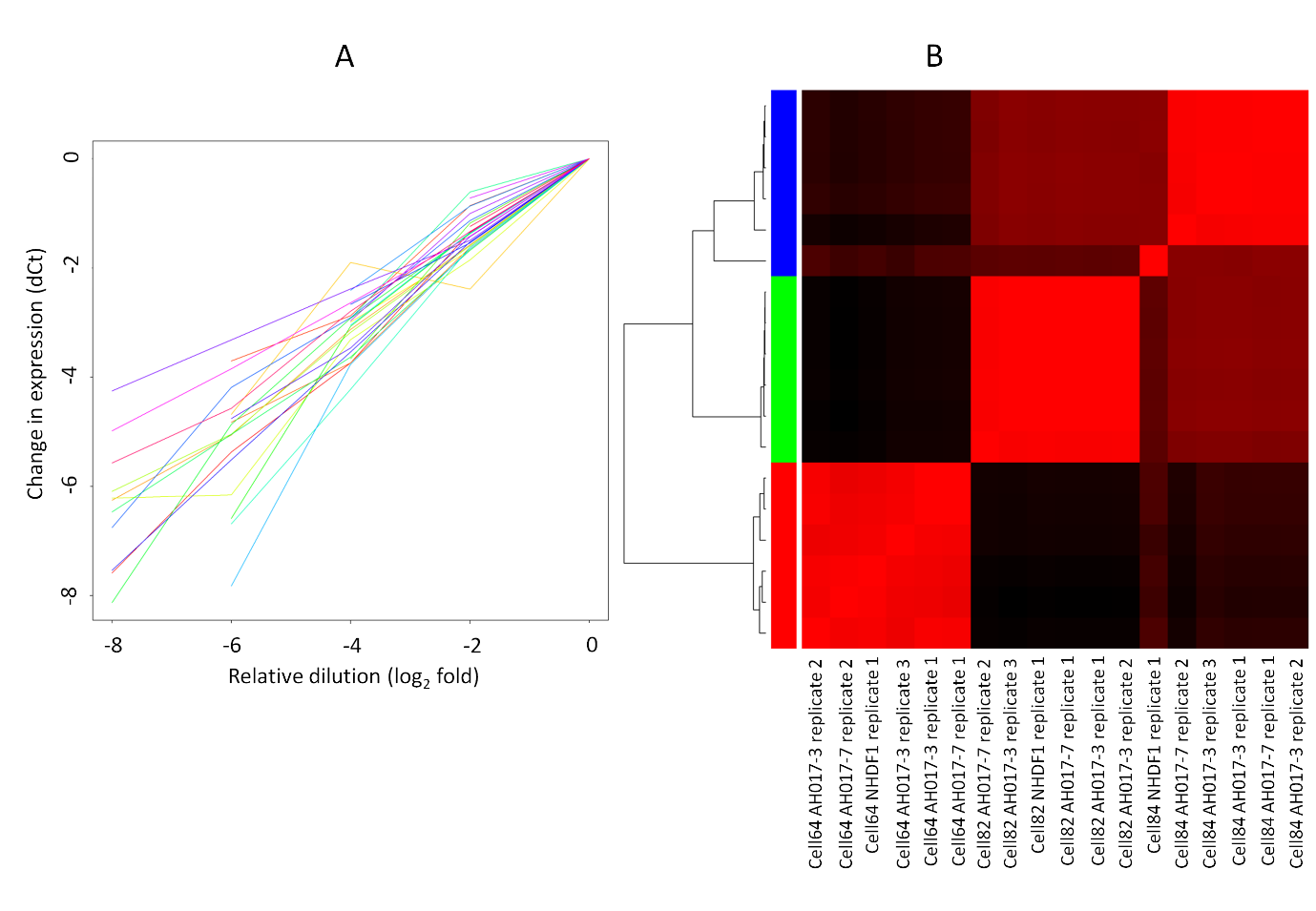


**Supplementary Figure 7 Biomark dilution series and inter-chip cDNA technical replicate correlation.** (A) Ct values with different dilutions of amplified single cell cDNA scaled to the maximum expression value for each gene. (B) Pairwise correlation between single cell cDNA technical replicates between chips. Vertical colour bar denotes technical replicate identity (red = cell 64; green = cell 82; and red = cell 84). The heatmap scale is from r = 0 (black) to r = 1 (red). It is apparent that cell 84 on the NHDF1 replicate 1 was mispipetted since the other internal controls on this chip worked well.
