# Supplementary material for: Assessing similarity to primary tissue and cortical layer identity in induced pluripotent stem cell-derived cortical neurons through single-cell transcriptomics
Source: Hum Mol Genet. 2016 Jan 5;25(5):989–1000. doi: 10.1093/hmg/ddv637 (PMC4754051; doi:10.1093/hmg/ddv637)
Supplement: Supplementary Data [file supp_ddv637_ddv637supp_tables.docx]

**Single cell RT-qPCR analysis of induced pluripotent stem cell-derived cortical neurons reveals frequent dual layer identity**

**Supplementary table 1 Number of cells analysed from each line**

| **Cell line** | **Age (days post-neural induction)** | **Number of cells / neurons** |
| --- | --- | --- |
| AH017-3 | 81 | 109 / 102 |
| AH017-3 | 180 | 69 / 66 |
| AH017-7 | 81 | 153 / 137 |
| NHDF1 | 81 | 75 / 75 |

**Supplementary table 2 Gene ontology enrichments for differentially expressed genes.** In each case, the cell type in which the gene set is more highly expressed is shown in the first column. Terms are ordered by enrichment.

| High cell type | Low cell type | Term | Fold Enrichment | Benjamini_FDR |
| --- | --- | --- | --- | --- |
| iPSC-derived cortical neurons | Adult cortical neurons | Transcription / Cell division and chromosome partitioning | 6.18 | 7.81E-03 |
| iPSC-derived cortical neurons | Adult cortical neurons | IPR017970:Homeobox, conserved site | 6.01 | 2.90E-02 |
| iPSC-derived cortical neurons | Adult cortical neurons | Homeobox | 5.92 | 1.99E-02 |
| iPSC-derived cortical neurons | Adult cortical neurons | SM00389:HOX | 5.40 | 2.86E-02 |
| iPSC-derived cortical neurons | Adult cortical neurons | neurogenesis | 4.16 | 1.50E-02 |
| iPSC-derived cortical neurons | Adult cortical neurons | GO:0040008~regulation of growth | 3.24 | 1.53E-02 |
| iPSC-derived cortical neurons | Adult cortical neurons | GO:0030182~neuron differentiation | 3.02 | 5.68E-03 |
| iPSC-derived cortical neurons | Adult cortical neurons | developmental protein | 2.92 | 5.94E-04 |
| iPSC-derived cortical neurons | Adult cortical neurons | GO:0006928~cell motion | 2.76 | 2.13E-02 |
| iPSC-derived cortical neurons | Adult cortical neurons | differentiation | 2.72 | 3.32E-02 |
| iPSC-derived cortical neurons | Adult cortical neurons | activator | 2.39 | 2.04E-02 |
| iPSC-derived cortical neurons | Adult cortical neurons | Secreted | 2.08 | 3.09E-02 |
| Adult cortical neurons | iPSC-derived cortical neurons | GO:0015085~calcium ion transmembrane transporter activity | 6.46 | 8.22E-03 |
| Adult cortical neurons | iPSC-derived cortical neurons | GO:0030672~synaptic vesicle membrane | 5.70 | 2.52E-05 |
| Adult cortical neurons | iPSC-derived cortical neurons | sushi | 4.58 | 7.03E-03 |
| Adult cortical neurons | iPSC-derived cortical neurons | GO:0008021~synaptic vesicle | 4.04 | 3.28E-08 |
| Adult cortical neurons | iPSC-derived cortical neurons | calcium transport | 3.60 | 3.13E-05 |
| Adult cortical neurons | iPSC-derived cortical neurons | GO:0015662~ATPase activity, coupled to transmembrane movement of ions, phosphorylative mechanism | 3.59 | 7.55E-04 |
| Adult cortical neurons | iPSC-derived cortical neurons | epilepsy | 3.55 | 4.58E-04 |
| Adult cortical neurons | iPSC-derived cortical neurons | GO:0019829~cation-transporting ATPase activity | 3.48 | 6.80E-03 |
| Adult cortical neurons | iPSC-derived cortical neurons | GO:0042734~presynaptic membrane | 3.46 | 3.71E-02 |
| Adult cortical neurons | iPSC-derived cortical neurons | potassium transport | 3.46 | 5.06E-05 |
| Adult cortical neurons | iPSC-derived cortical neurons | voltage-gated channel | 3.43 | 2.78E-06 |
| Adult cortical neurons | iPSC-derived cortical neurons | postsynaptic cell membrane | 3.43 | 2.78E-06 |
| Adult cortical neurons | iPSC-derived cortical neurons | hsa04730:Long-term depression | 3.32 | 5.47E-04 |
| Adult cortical neurons | iPSC-derived cortical neurons | GO:0030665~clathrin coated vesicle membrane | 3.27 | 5.68E-04 |
| Adult cortical neurons | iPSC-derived cortical neurons | hsa04930:Type II diabetes mellitus | 3.26 | 2.50E-02 |
| Adult cortical neurons | iPSC-derived cortical neurons | GO:0044456~synapse part | 3.25 | 1.24E-16 |
| Adult cortical neurons | iPSC-derived cortical neurons | synapse | 3.19 | 1.43E-11 |
| Adult cortical neurons | iPSC-derived cortical neurons | GO:0022843~voltage-gated cation channel activity | 3.18 | 3.04E-05 |
| Adult cortical neurons | iPSC-derived cortical neurons | hsa04720:Long-term potentiation | 3.16 | 4.32E-04 |
| Adult cortical neurons | iPSC-derived cortical neurons | GO:0042625~ATPase activity, coupled to transmembrane movement of ions | 3.11 | 3.19E-04 |
| Adult cortical neurons | iPSC-derived cortical neurons | potassium | 3.08 | 1.96E-04 |
| Adult cortical neurons | iPSC-derived cortical neurons | GO:0045211~postsynaptic membrane | 3.00 | 4.29E-06 |
| Adult cortical neurons | iPSC-derived cortical neurons | GO:0034703~cation channel complex | 2.97 | 3.28E-05 |
| Adult cortical neurons | iPSC-derived cortical neurons | GO:0014069~postsynaptic density | 2.90 | 9.63E-04 |
| Adult cortical neurons | iPSC-derived cortical neurons | Sodium transport | 2.88 | 4.95E-04 |
| Adult cortical neurons | iPSC-derived cortical neurons | GO:0030955~potassium ion binding | 2.86 | 1.10E-03 |
| Adult cortical neurons | iPSC-derived cortical neurons | GO:0008287~protein serine/threonine phosphatase complex | 2.84 | 1.81E-02 |
| Adult cortical neurons | iPSC-derived cortical neurons | Sodium | 2.84 | 6.07E-04 |
| Adult cortical neurons | iPSC-derived cortical neurons | GO:0006816~calcium ion transport | 2.81 | 2.00E-04 |
| Adult cortical neurons | iPSC-derived cortical neurons | GO:0030136~clathrin-coated vesicle | 2.80 | 2.05E-06 |
| Adult cortical neurons | iPSC-derived cortical neurons | GO:0003001~generation of a signal involved in cell-cell signaling | 2.80 | 2.79E-02 |
| Adult cortical neurons | iPSC-derived cortical neurons | GO:0005244~voltage-gated ion channel activity | 2.74 | 4.86E-05 |
| Adult cortical neurons | iPSC-derived cortical neurons | GO:0022832~voltage-gated channel activity | 2.74 | 4.86E-05 |
| Adult cortical neurons | iPSC-derived cortical neurons | GO:0045202~synapse | 2.74 | 2.63E-16 |
| Adult cortical neurons | iPSC-derived cortical neurons | GO:0006813~potassium ion transport | 2.71 | 8.16E-04 |
| Adult cortical neurons | iPSC-derived cortical neurons | GO:0005249~voltage-gated potassium channel activity | 2.71 | 2.66E-02 |
| Adult cortical neurons | iPSC-derived cortical neurons | GO:0006836~neurotransmitter transport | 2.69 | 2.74E-02 |
| Adult cortical neurons | iPSC-derived cortical neurons | hsa04912:GnRH signaling pathway | 2.68 | 1.49E-03 |
| Adult cortical neurons | iPSC-derived cortical neurons | GO:0031402~sodium ion binding | 2.68 | 2.74E-03 |
| Adult cortical neurons | iPSC-derived cortical neurons | GO:0031420~alkali metal ion binding | 2.64 | 2.86E-05 |
| Adult cortical neurons | iPSC-derived cortical neurons | GO:0055065~metal ion homeostasis | 2.61 | 9.85E-04 |
| Adult cortical neurons | iPSC-derived cortical neurons | GO:0034702~ion channel complex | 2.61 | 2.19E-05 |
| Adult cortical neurons | iPSC-derived cortical neurons | GO:0006754~ATP biosynthetic process | 2.58 | 1.00E-02 |
| Adult cortical neurons | iPSC-derived cortical neurons | ionic channel | 2.56 | 2.18E-06 |
| Adult cortical neurons | iPSC-derived cortical neurons | hsa04020:Calcium signaling pathway | 2.55 | 9.29E-04 |
| Adult cortical neurons | iPSC-derived cortical neurons | hsa04270:Vascular smooth muscle contraction | 2.55 | 6.71E-03 |
| Adult cortical neurons | iPSC-derived cortical neurons | GO:0006814~sodium ion transport | 2.51 | 1.01E-02 |
| Adult cortical neurons | iPSC-derived cortical neurons | GO:0006875~cellular metal ion homeostasis | 2.51 | 4.03E-03 |
| Adult cortical neurons | iPSC-derived cortical neurons | GO:0055074~calcium ion homeostasis | 2.50 | 5.57E-03 |
| Adult cortical neurons | iPSC-derived cortical neurons | GO:0005267~potassium channel activity | 2.49 | 1.98E-02 |
| Adult cortical neurons | iPSC-derived cortical neurons | GO:0043492~ATPase activity, coupled to movement of substances | 2.48 | 3.64E-03 |
| Adult cortical neurons | iPSC-derived cortical neurons | GO:0030659~cytoplasmic vesicle membrane | 2.47 | 1.35E-04 |
| Adult cortical neurons | iPSC-derived cortical neurons | GO:0006874~cellular calcium ion homeostasis | 2.46 | 1.02E-02 |
| Adult cortical neurons | iPSC-derived cortical neurons | GO:0012506~vesicle membrane | 2.44 | 8.93E-05 |
| Adult cortical neurons | iPSC-derived cortical neurons | GO:0030135~coated vesicle | 2.43 | 1.91E-05 |
| Adult cortical neurons | iPSC-derived cortical neurons | GO:0030662~coated vesicle membrane | 2.42 | 1.12E-02 |
| Adult cortical neurons | iPSC-derived cortical neurons | GO:0022836~gated channel activity | 2.42 | 2.04E-05 |
| Adult cortical neurons | iPSC-derived cortical neurons | GO:0055080~cation homeostasis | 2.42 | 5.23E-05 |
| Adult cortical neurons | iPSC-derived cortical neurons | GO:0016820~hydrolase activity, acting on acid anhydrides, catalyzing transmembrane movement of substances | 2.40 | 8.03E-03 |
| Adult cortical neurons | iPSC-derived cortical neurons | GO:0042626~ATPase activity, coupled to transmembrane movement of substances | 2.40 | 8.03E-03 |
| Adult cortical neurons | iPSC-derived cortical neurons | GO:0015672~monovalent inorganic cation transport | 2.37 | 4.37E-06 |
| Adult cortical neurons | iPSC-derived cortical neurons | GO:0007268~synaptic transmission | 2.36 | 2.41E-06 |
| Adult cortical neurons | iPSC-derived cortical neurons | GO:0015674~di-, tri-valent inorganic cation transport | 2.36 | 3.23E-03 |
| Adult cortical neurons | iPSC-derived cortical neurons | GO:0009206~purine ribonucleoside triphosphate biosynthetic process | 2.33 | 3.31E-02 |
| Adult cortical neurons | iPSC-derived cortical neurons | GO:0005261~cation channel activity | 2.32 | 1.95E-04 |
| Adult cortical neurons | iPSC-derived cortical neurons | GO:0009201~ribonucleoside triphosphate biosynthetic process | 2.30 | 3.77E-02 |
| Adult cortical neurons | iPSC-derived cortical neurons | GO:0009145~purine nucleoside triphosphate biosynthetic process | 2.30 | 3.77E-02 |
| Adult cortical neurons | iPSC-derived cortical neurons | GO:0055066~di-, tri-valent inorganic cation homeostasis | 2.26 | 4.04E-03 |
| Adult cortical neurons | iPSC-derived cortical neurons | GO:0015405~P-P-bond-hydrolysis-driven transmembrane transporter activity | 2.26 | 9.07E-03 |
| Adult cortical neurons | iPSC-derived cortical neurons | GO:0015399~primary active transmembrane transporter activity | 2.26 | 9.07E-03 |
| Adult cortical neurons | iPSC-derived cortical neurons | GO:0030003~cellular cation homeostasis | 2.21 | 4.00E-03 |
| Adult cortical neurons | iPSC-derived cortical neurons | GO:0019226~transmission of nerve impulse | 2.21 | 4.33E-06 |
| Adult cortical neurons | iPSC-derived cortical neurons | GO:0005516~calmodulin binding | 2.21 | 9.38E-03 |
| Adult cortical neurons | iPSC-derived cortical neurons | GO:0046034~ATP metabolic process | 2.21 | 4.79E-02 |
| Adult cortical neurons | iPSC-derived cortical neurons | hsa04540:Gap junction | 2.20 | 4.52E-02 |
| Adult cortical neurons | iPSC-derived cortical neurons | cell junction | 2.20 | 3.26E-07 |
| Adult cortical neurons | iPSC-derived cortical neurons | GO:0007626~locomotory behavior | 2.18 | 5.77E-03 |
| Adult cortical neurons | iPSC-derived cortical neurons | GO:0030005~cellular di-, tri-valent inorganic cation homeostasis | 2.18 | 1.65E-02 |
| Adult cortical neurons | iPSC-derived cortical neurons | GO:0005216~ion channel activity | 2.17 | 5.61E-05 |
| Adult cortical neurons | iPSC-derived cortical neurons | GO:0030001~metal ion transport | 2.16 | 1.58E-06 |
| Adult cortical neurons | iPSC-derived cortical neurons | GO:0046873~metal ion transmembrane transporter activity | 2.15 | 1.61E-04 |
| Adult cortical neurons | iPSC-derived cortical neurons | ion transport | 2.15 | 4.83E-08 |
| Adult cortical neurons | iPSC-derived cortical neurons | GO:0022838~substrate specific channel activity | 2.14 | 6.79E-05 |
| Adult cortical neurons | iPSC-derived cortical neurons | GO:0030424~axon | 2.13 | 9.98E-04 |
| Adult cortical neurons | iPSC-derived cortical neurons | GO:0022803~passive transmembrane transporter activity | 2.12 | 8.32E-05 |
| Adult cortical neurons | iPSC-derived cortical neurons | GO:0015267~channel activity | 2.12 | 8.32E-05 |
| Adult cortical neurons | iPSC-derived cortical neurons | GO:0044433~cytoplasmic vesicle part | 2.10 | 1.49E-03 |
| Adult cortical neurons | iPSC-derived cortical neurons | palmitate | 2.10 | 1.18E-02 |
| Adult cortical neurons | iPSC-derived cortical neurons | GO:0006812~cation transport | 2.02 | 1.31E-06 |
| Adult cortical neurons | iPSC-derived cortical neurons | GO:0050801~ion homeostasis | 2.02 | 3.56E-04 |
| Adult cortical neurons | iPSC-derived cortical neurons | GO:0032940~secretion by cell | 2.01 | 3.36E-02 |
| Adult cortical neurons | iPSC-derived cortical neurons | GO:0006164~purine nucleotide biosynthetic process | 2.00 | 4.95E-02 |
| Adult cortical neurons | iPSC-derived cortical neurons | tpr repeat | 1.99 | 1.01E-02 |
| Adult cortical neurons | iPSC-derived cortical neurons | GO:0043005~neuron projection | 1.99 | 5.65E-06 |
| Adult cortical neurons | iPSC-derived cortical neurons | GO:0007610~behavior | 1.97 | 3.63E-04 |
| Adult cortical neurons | iPSC-derived cortical neurons | GO:0005083~small GTPase regulator activity | 1.97 | 4.84E-04 |
| Adult cortical neurons | iPSC-derived cortical neurons | GO:0006811~ion transport | 1.95 | 9.44E-08 |
| Adult cortical neurons | iPSC-derived cortical neurons | GO:0030054~cell junction | 1.94 | 6.79E-07 |
| Adult cortical neurons | iPSC-derived cortical neurons | GO:0055082~cellular chemical homeostasis | 1.93 | 3.30E-03 |
| Adult cortical neurons | iPSC-derived cortical neurons | GO:0006873~cellular ion homeostasis | 1.92 | 4.16E-03 |
| Adult cortical neurons | iPSC-derived cortical neurons | GO:0007267~cell-cell signaling | 1.88 | 2.16E-04 |
| Adult cortical neurons | iPSC-derived cortical neurons | calcium | 1.86 | 1.21E-06 |
| Adult cortical neurons | iPSC-derived cortical neurons | cell adhesion | 1.84 | 2.69E-03 |
| Adult cortical neurons | iPSC-derived cortical neurons | cytoplasmic vesicle | 1.79 | 1.92E-02 |
| Adult cortical neurons | iPSC-derived cortical neurons | Immunoglobulin domain | 1.77 | 1.51E-02 |
| Adult cortical neurons | iPSC-derived cortical neurons | GO:0050877~neurological system process | 1.77 | 1.58E-05 |
| Adult cortical neurons | iPSC-derived cortical neurons | compositionally biased region:Ser-rich | 1.72 | 6.83E-03 |
| Adult cortical neurons | iPSC-derived cortical neurons | GO:0030695~GTPase regulator activity | 1.72 | 1.43E-03 |
| Adult cortical neurons | iPSC-derived cortical neurons | GO:0048878~chemical homeostasis | 1.71 | 1.07E-02 |
| Adult cortical neurons | iPSC-derived cortical neurons | GO:0005509~calcium ion binding | 1.71 | 2.25E-05 |
| Adult cortical neurons | iPSC-derived cortical neurons | GO:0048666~neuron development | 1.70 | 2.40E-02 |
| Adult cortical neurons | iPSC-derived cortical neurons | GO:0060589~nucleoside-triphosphatase regulator activity | 1.68 | 2.61E-03 |
| Adult cortical neurons | iPSC-derived cortical neurons | cell membrane | 1.65 | 1.17E-08 |
| Adult cortical neurons | iPSC-derived cortical neurons | lipoprotein | 1.61 | 2.89E-03 |
| Adult cortical neurons | iPSC-derived cortical neurons | GO:0030182~neuron differentiation | 1.60 | 3.23E-02 |
| Adult cortical neurons | iPSC-derived cortical neurons | golgi apparatus | 1.57 | 1.11E-03 |
| Adult cortical neurons | iPSC-derived cortical neurons | GO:0019898~extrinsic to membrane | 1.57 | 3.18E-03 |
| Adult cortical neurons | iPSC-derived cortical neurons | GO:0016023~cytoplasmic membrane-bounded vesicle | 1.56 | 2.17E-03 |
| Adult cortical neurons | iPSC-derived cortical neurons | GO:0007155~cell adhesion | 1.55 | 1.95E-02 |
| Adult cortical neurons | iPSC-derived cortical neurons | GO:0022610~biological adhesion | 1.55 | 1.95E-02 |
| Adult cortical neurons | iPSC-derived cortical neurons | GO:0031988~membrane-bounded vesicle | 1.55 | 1.88E-03 |
| Adult cortical neurons | iPSC-derived cortical neurons | GO:0031982~vesicle | 1.53 | 9.37E-04 |
| Adult cortical neurons | iPSC-derived cortical neurons | GO:0042995~cell projection | 1.51 | 1.48E-03 |
| Adult cortical neurons | iPSC-derived cortical neurons | GO:0008092~cytoskeletal protein binding | 1.48 | 3.64E-02 |
| Adult cortical neurons | iPSC-derived cortical neurons | GO:0031410~cytoplasmic vesicle | 1.48 | 4.26E-03 |
| Adult cortical neurons | iPSC-derived cortical neurons | GO:0044459~plasma membrane part | 1.46 | 1.57E-07 |
| Adult cortical neurons | iPSC-derived cortical neurons | GO:0005887~integral to plasma membrane | 1.45 | 2.71E-03 |
| Adult cortical neurons | iPSC-derived cortical neurons | GO:0005886~plasma membrane | 1.45 | 1.34E-12 |
| Adult cortical neurons | iPSC-derived cortical neurons | topological domain:Extracellular | 1.43 | 8.83E-04 |
| Adult cortical neurons | iPSC-derived cortical neurons | GO:0031226~intrinsic to plasma membrane | 1.41 | 6.58E-03 |
| Adult cortical neurons | iPSC-derived cortical neurons | topological domain:Cytoplasmic | 1.38 | 1.56E-04 |
| Adult cortical neurons | iPSC-derived cortical neurons | GO:0005794~Golgi apparatus | 1.37 | 6.14E-03 |
| Adult cortical neurons | iPSC-derived cortical neurons | transport | 1.37 | 3.16E-04 |
| Adult cortical neurons | iPSC-derived cortical neurons | glycoprotein | 1.32 | 2.92E-05 |
| Adult cortical neurons | iPSC-derived cortical neurons | glycosylation site:N-linked (GlcNAc...) | 1.30 | 2.43E-03 |
| Adult cortical neurons | iPSC-derived cortical neurons | membrane | 1.29 | 6.66E-10 |
| Adult cortical neurons | iPSC-derived cortical neurons | transmembrane region | 1.25 | 8.78E-04 |
| Adult cortical neurons | iPSC-derived cortical neurons | transmembrane | 1.25 | 4.40E-05 |
| Adult cortical neurons | iPSC-derived cortical neurons | signal | 1.23 | 2.91E-02 |
| Adult cortical neurons | iPSC-derived cortical neurons | GO:0031224~intrinsic to membrane | 1.19 | 1.37E-04 |
| Adult cortical neurons | iPSC-derived cortical neurons | alternative splicing | 1.17 | 9.64E-08 |
| Adult cortical neurons | iPSC-derived cortical neurons | GO:0016021~integral to membrane | 1.17 | 1.31E-03 |
| Adult cortical neurons | iPSC-derived cortical neurons | splice variant | 1.16 | 4.21E-05 |
| Adult cortical neurons | iPSC-derived cortical neurons | phosphoprotein | 1.09 | 7.88E-03 |
| iPSC-derived cortical neurons | Fetal quiescent | gluconeogenesis | 11.75 | 3.47E-04 |
| iPSC-derived cortical neurons | Fetal quiescent | h_glycolysisPathway:Glycolysis Pathway | 9.18 | 1.62E-02 |
| iPSC-derived cortical neurons | Fetal quiescent | glycolysis | 5.51 | 6.07E-04 |
| iPSC-derived cortical neurons | Fetal quiescent | GO:0006096~glycolysis | 4.85 | 4.67E-03 |
| iPSC-derived cortical neurons | Fetal quiescent | GO:0006007~glucose catabolic process | 4.40 | 2.92E-03 |
| iPSC-derived cortical neurons | Fetal quiescent | GO:0009063~cellular amino acid catabolic process | 4.35 | 9.98E-03 |
| iPSC-derived cortical neurons | Fetal quiescent | GO:0046365~monosaccharide catabolic process | 4.27 | 1.38E-03 |
| iPSC-derived cortical neurons | Fetal quiescent | GO:0046164~alcohol catabolic process | 4.22 | 9.55E-04 |
| iPSC-derived cortical neurons | Fetal quiescent | GO:0009310~amine catabolic process | 4.18 | 7.19E-03 |
| iPSC-derived cortical neurons | Fetal quiescent | GO:0019320~hexose catabolic process | 4.16 | 2.97E-03 |
| iPSC-derived cortical neurons | Fetal quiescent | GO:0046395~carboxylic acid catabolic process | 4.04 | 2.45E-03 |
| iPSC-derived cortical neurons | Fetal quiescent | GO:0016054~organic acid catabolic process | 4.04 | 2.45E-03 |
| iPSC-derived cortical neurons | Fetal quiescent | hsa00010:Glycolysis / Gluconeogenesis | 4.00 | 1.45E-02 |
| iPSC-derived cortical neurons | Fetal quiescent | GO:0044275~cellular carbohydrate catabolic process | 3.77 | 3.69E-03 |
| iPSC-derived cortical neurons | Fetal quiescent | GO:0048770~pigment granule | 3.24 | 5.47E-03 |
| iPSC-derived cortical neurons | Fetal quiescent | GO:0042470~melanosome | 3.24 | 5.47E-03 |
| iPSC-derived cortical neurons | Fetal quiescent | GO:0016052~carbohydrate catabolic process | 3.19 | 1.74E-02 |
| iPSC-derived cortical neurons | Fetal quiescent | GO:0006006~glucose metabolic process | 2.91 | 5.72E-03 |
| iPSC-derived cortical neurons | Fetal quiescent | IPR016040:NAD(P)-binding domain | 2.83 | 2.71E-02 |
| iPSC-derived cortical neurons | Fetal quiescent | GO:0019318~hexose metabolic process | 2.63 | 6.54E-03 |
| iPSC-derived cortical neurons | Fetal quiescent | nad | 2.52 | 8.32E-03 |
| iPSC-derived cortical neurons | Fetal quiescent | GO:0005996~monosaccharide metabolic process | 2.37 | 1.75E-02 |
| iPSC-derived cortical neurons | Fetal quiescent | GO:0006091~generation of precursor metabolites and energy | 2.37 | 1.42E-03 |
| iPSC-derived cortical neurons | Fetal quiescent | oxidoreductase | 2.07 | 5.88E-04 |
| iPSC-derived cortical neurons | Fetal quiescent | GO:0055114~oxidation reduction | 1.93 | 1.72E-03 |
| iPSC-derived cortical neurons | Fetal quiescent | mitochondrion | 1.71 | 5.62E-04 |
| iPSC-derived cortical neurons | Fetal quiescent | GO:0005739~mitochondrion | 1.61 | 1.76E-04 |
| iPSC-derived cortical neurons | Fetal quiescent | disease mutation | 1.55 | 1.19E-03 |
| iPSC-derived cortical neurons | Fetal quiescent | signal | 1.34 | 4.73E-02 |
| Fetal quiescent | iPSC-derived cortical neurons | neurogenesis | 10.07 | 4.32E-06 |
| Fetal quiescent | iPSC-derived cortical neurons | hsa03010:Ribosome | 8.45 | 1.06E-03 |
| Fetal quiescent | iPSC-derived cortical neurons | ribosome | 8.39 | 2.49E-02 |
| Fetal quiescent | iPSC-derived cortical neurons | GO:0022626~cytosolic ribosome | 7.90 | 3.90E-02 |
| Fetal quiescent | iPSC-derived cortical neurons | GO:0006414~translational elongation | 7.55 | 1.32E-02 |
| Fetal quiescent | iPSC-derived cortical neurons | differentiation | 5.82 | 2.19E-05 |
| Fetal quiescent | iPSC-derived cortical neurons | GO:0007409~axonogenesis | 5.48 | 2.45E-02 |
| Fetal quiescent | iPSC-derived cortical neurons | GO:0048812~neuron projection morphogenesis | 5.44 | 1.10E-02 |
| Fetal quiescent | iPSC-derived cortical neurons | GO:0048667~cell morphogenesis involved in neuron differentiation | 5.15 | 2.91E-02 |
| Fetal quiescent | iPSC-derived cortical neurons | GO:0031175~neuron projection development | 5.08 | 1.41E-02 |
| Fetal quiescent | iPSC-derived cortical neurons | GO:0016477~cell migration | 4.99 | 3.26E-02 |
| Fetal quiescent | iPSC-derived cortical neurons | GO:0030182~neuron differentiation | 4.87 | 5.43E-04 |
| Fetal quiescent | iPSC-derived cortical neurons | GO:0048858~cell projection morphogenesis | 4.64 | 2.72E-02 |
| Fetal quiescent | iPSC-derived cortical neurons | GO:0000904~cell morphogenesis involved in differentiation | 4.60 | 4.62E-02 |
| Fetal quiescent | iPSC-derived cortical neurons | ribosomal protein | 4.59 | 3.83E-02 |
| Fetal quiescent | iPSC-derived cortical neurons | GO:0048870~cell motility | 4.57 | 4.44E-02 |
| Fetal quiescent | iPSC-derived cortical neurons | GO:0051674~localization of cell | 4.57 | 4.44E-02 |
| Fetal quiescent | iPSC-derived cortical neurons | developmental protein | 4.45 | 6.54E-05 |
| Fetal quiescent | iPSC-derived cortical neurons | GO:0048666~neuron development | 4.44 | 1.39E-02 |
| Fetal quiescent | iPSC-derived cortical neurons | GO:0032990~cell part morphogenesis | 4.38 | 3.03E-02 |
| Fetal quiescent | iPSC-derived cortical neurons | GO:0006928~cell motion | 4.25 | 8.00E-03 |
| Fetal quiescent | iPSC-derived cortical neurons | chromosomal rearrangement | 4.03 | 4.24E-02 |
| Fetal quiescent | iPSC-derived cortical neurons | ribonucleoprotein | 3.75 | 3.88E-02 |
| Fetal quiescent | iPSC-derived cortical neurons | GO:0003700~transcription factor activity | 2.94 | 2.38E-02 |
| Fetal quiescent | iPSC-derived cortical neurons | GO:0030528~transcription regulator activity | 2.17 | 4.02E-02 |
| Fetal quiescent | iPSC-derived cortical neurons | phosphoprotein | 1.32 | 2.56E-02 |
| iPSC-derived cortical neurons | Fetal replicating | gluconeogenesis | 10.15 | 4.18E-02 |
| iPSC-derived cortical neurons | Fetal replicating | GO:0033176~proton-transporting V-type ATPase complex | 7.30 | 2.08E-02 |
| iPSC-derived cortical neurons | Fetal replicating | ubiquinone | 7.06 | 6.09E-03 |
| iPSC-derived cortical neurons | Fetal replicating | Hydrogen ion transport | 6.15 | 1.87E-03 |
| iPSC-derived cortical neurons | Fetal replicating | GO:0016469~proton-transporting two-sector ATPase complex | 5.91 | 4.44E-04 |
| iPSC-derived cortical neurons | Fetal replicating | glycolysis | 5.71 | 7.59E-03 |
| iPSC-derived cortical neurons | Fetal replicating | GO:0015985~energy coupled proton transport, down electrochemical gradient | 5.63 | 1.80E-02 |
| iPSC-derived cortical neurons | Fetal replicating | GO:0015986~ATP synthesis coupled proton transport | 5.63 | 1.80E-02 |
| iPSC-derived cortical neurons | Fetal replicating | GO:0006096~glycolysis | 5.54 | 1.10E-02 |
| iPSC-derived cortical neurons | Fetal replicating | respiratory chain | 5.54 | 1.22E-04 |
| iPSC-derived cortical neurons | Fetal replicating | GO:0015078~hydrogen ion transmembrane transporter activity | 5.28 | 2.21E-05 |
| iPSC-derived cortical neurons | Fetal replicating | hsa05110:Vibrio cholerae infection | 5.20 | 2.85E-04 |
| iPSC-derived cortical neurons | Fetal replicating | hsa00051:Fructose and mannose metabolism | 5.14 | 1.13E-02 |
| iPSC-derived cortical neurons | Fetal replicating | GO:0050136~NADH dehydrogenase (quinone) activity | 5.11 | 3.04E-02 |
| iPSC-derived cortical neurons | Fetal replicating | GO:0008137~NADH dehydrogenase (ubiquinone) activity | 5.11 | 3.04E-02 |
| iPSC-derived cortical neurons | Fetal replicating | GO:0003954~NADH dehydrogenase activity | 5.11 | 3.04E-02 |
| iPSC-derived cortical neurons | Fetal replicating | GO:0015077~monovalent inorganic cation transmembrane transporter activity | 5.10 | 1.04E-05 |
| iPSC-derived cortical neurons | Fetal replicating | GO:0006120~mitochondrial electron transport, NADH to ubiquinone | 4.99 | 3.12E-02 |
| iPSC-derived cortical neurons | Fetal replicating | GO:0006119~oxidative phosphorylation | 4.97 | 8.80E-06 |
| iPSC-derived cortical neurons | Fetal replicating | GO:0016655~oxidoreductase activity, acting on NADH or NADPH, quinone or similar compound as acceptor | 4.97 | 1.73E-02 |
| iPSC-derived cortical neurons | Fetal replicating | GO:0042775~mitochondrial ATP synthesis coupled electron transport | 4.85 | 1.08E-02 |
| iPSC-derived cortical neurons | Fetal replicating | GO:0042773~ATP synthesis coupled electron transport | 4.85 | 1.08E-02 |
| iPSC-derived cortical neurons | Fetal replicating | GO:0015992~proton transport | 4.85 | 2.01E-02 |
| iPSC-derived cortical neurons | Fetal replicating | GO:0034220~ion transmembrane transport | 4.85 | 3.33E-02 |
| iPSC-derived cortical neurons | Fetal replicating | GO:0030964~NADH dehydrogenase complex | 4.83 | 9.93E-03 |
| iPSC-derived cortical neurons | Fetal replicating | GO:0005747~mitochondrial respiratory chain complex I | 4.83 | 9.93E-03 |
| iPSC-derived cortical neurons | Fetal replicating | GO:0045271~respiratory chain complex I | 4.83 | 9.93E-03 |
| iPSC-derived cortical neurons | Fetal replicating | hsa00010:Glycolysis / Gluconeogenesis | 4.82 | 2.94E-03 |
| iPSC-derived cortical neurons | Fetal replicating | GO:0006818~hydrogen transport | 4.62 | 2.64E-02 |
| iPSC-derived cortical neurons | Fetal replicating | hsa00190:Oxidative phosphorylation | 4.61 | 1.96E-08 |
| iPSC-derived cortical neurons | Fetal replicating | GO:0005746~mitochondrial respiratory chain | 4.47 | 2.08E-03 |
| iPSC-derived cortical neurons | Fetal replicating | GO:0016651~oxidoreductase activity, acting on NADH or NADPH | 4.35 | 2.31E-03 |
| iPSC-derived cortical neurons | Fetal replicating | GO:0006007~glucose catabolic process | 4.31 | 3.23E-02 |
| iPSC-derived cortical neurons | Fetal replicating | hsa05012:Parkinson's disease | 4.29 | 2.97E-07 |
| iPSC-derived cortical neurons | Fetal replicating | electron transport | 4.28 | 3.82E-04 |
| iPSC-derived cortical neurons | Fetal replicating | GO:0070469~respiratory chain | 4.24 | 1.73E-03 |
| iPSC-derived cortical neurons | Fetal replicating | GO:0022904~respiratory electron transport chain | 4.10 | 2.89E-02 |
| iPSC-derived cortical neurons | Fetal replicating | GO:0022890~inorganic cation transmembrane transporter activity | 4.09 | 2.72E-05 |
| iPSC-derived cortical neurons | Fetal replicating | hsa05130:Pathogenic Escherichia coli infection | 4.00 | 2.16E-02 |
| iPSC-derived cortical neurons | Fetal replicating | GO:0046034~ATP metabolic process | 3.78 | 7.56E-03 |
| iPSC-derived cortical neurons | Fetal replicating | GO:0046164~alcohol catabolic process | 3.74 | 4.01E-02 |
| iPSC-derived cortical neurons | Fetal replicating | GO:0022900~electron transport chain | 3.70 | 6.62E-03 |
| iPSC-derived cortical neurons | Fetal replicating | GO:0009205~purine ribonucleoside triphosphate metabolic process | 3.58 | 6.16E-03 |
| iPSC-derived cortical neurons | Fetal replicating | GO:0009199~ribonucleoside triphosphate metabolic process | 3.54 | 5.91E-03 |
| iPSC-derived cortical neurons | Fetal replicating | GO:0044455~mitochondrial membrane part | 3.50 | 4.48E-04 |
| iPSC-derived cortical neurons | Fetal replicating | GO:0006091~generation of precursor metabolites and energy | 3.50 | 7.50E-09 |
| iPSC-derived cortical neurons | Fetal replicating | GO:0006754~ATP biosynthetic process | 3.47 | 4.03E-02 |
| iPSC-derived cortical neurons | Fetal replicating | GO:0009206~purine ribonucleoside triphosphate biosynthetic process | 3.41 | 3.31E-02 |
| iPSC-derived cortical neurons | Fetal replicating | GO:0009144~purine nucleoside triphosphate metabolic process | 3.40 | 7.63E-03 |
| iPSC-derived cortical neurons | Fetal replicating | GO:0009201~ribonucleoside triphosphate biosynthetic process | 3.36 | 3.16E-02 |
| iPSC-derived cortical neurons | Fetal replicating | GO:0009145~purine nucleoside triphosphate biosynthetic process | 3.36 | 3.16E-02 |
| iPSC-derived cortical neurons | Fetal replicating | GO:0045333~cellular respiration | 3.35 | 2.77E-02 |
| iPSC-derived cortical neurons | Fetal replicating | GO:0009141~nucleoside triphosphate metabolic process | 3.23 | 1.15E-02 |
| iPSC-derived cortical neurons | Fetal replicating | GO:0009142~nucleoside triphosphate biosynthetic process | 3.23 | 3.80E-02 |
| iPSC-derived cortical neurons | Fetal replicating | GO:0030424~axon | 3.21 | 4.65E-04 |
| iPSC-derived cortical neurons | Fetal replicating | mitochondrion inner membrane | 3.21 | 3.00E-04 |
| iPSC-derived cortical neurons | Fetal replicating | GO:0019717~synaptosome | 3.19 | 4.10E-02 |
| iPSC-derived cortical neurons | Fetal replicating | hsa05010:Alzheimer's disease | 3.18 | 1.09E-04 |
| iPSC-derived cortical neurons | Fetal replicating | GO:0009150~purine ribonucleotide metabolic process | 3.05 | 1.93E-02 |
| iPSC-derived cortical neurons | Fetal replicating | GO:0009152~purine ribonucleotide biosynthetic process | 3.02 | 4.08E-02 |
| iPSC-derived cortical neurons | Fetal replicating | GO:0009259~ribonucleotide metabolic process | 2.82 | 3.13E-02 |
| iPSC-derived cortical neurons | Fetal replicating | GO:0006164~purine nucleotide biosynthetic process | 2.80 | 3.98E-02 |
| iPSC-derived cortical neurons | Fetal replicating | GO:0048193~Golgi vesicle transport | 2.77 | 3.29E-02 |
| iPSC-derived cortical neurons | Fetal replicating | GO:0006163~purine nucleotide metabolic process | 2.71 | 1.89E-02 |
| iPSC-derived cortical neurons | Fetal replicating | hsa05016:Huntington's disease | 2.65 | 1.24E-03 |
| iPSC-derived cortical neurons | Fetal replicating | GO:0005743~mitochondrial inner membrane | 2.52 | 1.19E-03 |
| iPSC-derived cortical neurons | Fetal replicating | GO:0009165~nucleotide biosynthetic process | 2.51 | 4.55E-02 |
| iPSC-derived cortical neurons | Fetal replicating | GO:0043005~neuron projection | 2.39 | 4.10E-04 |
| iPSC-derived cortical neurons | Fetal replicating | GO:0019866~organelle inner membrane | 2.39 | 4.10E-04 |
| iPSC-derived cortical neurons | Fetal replicating | GO:0005625~soluble fraction | 2.31 | 7.57E-03 |
| iPSC-derived cortical neurons | Fetal replicating | GO:0031966~mitochondrial membrane | 2.28 | 6.24E-04 |
| iPSC-derived cortical neurons | Fetal replicating | GO:0005740~mitochondrial envelope | 2.20 | 5.56E-04 |
| iPSC-derived cortical neurons | Fetal replicating | GO:0016192~vesicle-mediated transport | 1.97 | 5.59E-03 |
| iPSC-derived cortical neurons | Fetal replicating | ion transport | 1.96 | 4.23E-02 |
| iPSC-derived cortical neurons | Fetal replicating | GO:0055114~oxidation reduction | 1.81 | 3.88E-02 |
| iPSC-derived cortical neurons | Fetal replicating | GO:0031967~organelle envelope | 1.80 | 3.07E-03 |
| iPSC-derived cortical neurons | Fetal replicating | GO:0031975~envelope | 1.79 | 2.94E-03 |
| iPSC-derived cortical neurons | Fetal replicating | protein transport | 1.79 | 3.86E-02 |
| iPSC-derived cortical neurons | Fetal replicating | GO:0044429~mitochondrial part | 1.77 | 5.14E-03 |
| iPSC-derived cortical neurons | Fetal replicating | GO:0042995~cell projection | 1.70 | 1.56E-02 |
| iPSC-derived cortical neurons | Fetal replicating | GO:0031090~organelle membrane | 1.67 | 4.10E-04 |
| iPSC-derived cortical neurons | Fetal replicating | transport | 1.66 | 2.15E-04 |
| iPSC-derived cortical neurons | Fetal replicating | GO:0005739~mitochondrion | 1.51 | 9.19E-03 |
| iPSC-derived cortical neurons | Fetal replicating | GO:0005829~cytosol | 1.39 | 3.62E-02 |
| iPSC-derived cortical neurons | Fetal replicating | acetylation | 1.29 | 2.80E-02 |
| Fetal replicating | iPSC-derived cortical neurons | 114.Genomic_reformatting_Brain_Ischemia | 30.75 | 1.13E-02 |
| Fetal replicating | iPSC-derived cortical neurons | GO:0048704~embryonic skeletal system morphogenesis | 16.29 | 4.78E-02 |
| Fetal replicating | iPSC-derived cortical neurons | GO:0030261~chromosome condensation | 16.29 | 4.78E-02 |
| Fetal replicating | iPSC-derived cortical neurons | cell cycle control | 13.73 | 4.66E-02 |
| Fetal replicating | iPSC-derived cortical neurons | GO:0051216~cartilage development | 12.64 | 1.79E-02 |
| Fetal replicating | iPSC-derived cortical neurons | stress response | 9.98 | 2.62E-02 |
| Fetal replicating | iPSC-derived cortical neurons | GO:0048705~skeletal system morphogenesis | 9.56 | 1.16E-02 |
| Fetal replicating | iPSC-derived cortical neurons | GO:0007059~chromosome segregation | 7.09 | 4.20E-02 |
| Fetal replicating | iPSC-derived cortical neurons | mitosis | 5.88 | 2.03E-03 |
| Fetal replicating | iPSC-derived cortical neurons | GO:0043566~structure-specific DNA binding | 5.60 | 2.24E-02 |
| Fetal replicating | iPSC-derived cortical neurons | GO:0043565~sequence-specific DNA binding | 5.52 | 5.83E-07 |
| Fetal replicating | iPSC-derived cortical neurons | cell division | 5.24 | 4.50E-04 |
| Fetal replicating | iPSC-derived cortical neurons | GO:0000122~negative regulation of transcription from RNA polymerase II promoter | 5.24 | 7.02E-04 |
| Fetal replicating | iPSC-derived cortical neurons | DNA binding | 5.12 | 1.10E-03 |
| Fetal replicating | iPSC-derived cortical neurons | GO:0007067~mitosis | 4.77 | 3.22E-03 |
| Fetal replicating | iPSC-derived cortical neurons | GO:0000280~nuclear division | 4.77 | 3.22E-03 |
| Fetal replicating | iPSC-derived cortical neurons | GO:0045944~positive regulation of transcription from RNA polymerase II promoter | 4.75 | 3.38E-04 |
| Fetal replicating | iPSC-derived cortical neurons | GO:0000087~M phase of mitotic cell cycle | 4.66 | 3.80E-03 |
| Fetal replicating | iPSC-derived cortical neurons | GO:0045892~negative regulation of transcription, DNA-dependent | 4.66 | 2.24E-04 |
| Fetal replicating | iPSC-derived cortical neurons | GO:0048285~organelle fission | 4.55 | 4.46E-03 |
| Fetal replicating | iPSC-derived cortical neurons | GO:0051253~negative regulation of RNA metabolic process | 4.54 | 2.41E-04 |
| Fetal replicating | iPSC-derived cortical neurons | cell cycle | 4.49 | 3.77E-05 |
| Fetal replicating | iPSC-derived cortical neurons | GO:0045893~positive regulation of transcription, DNA-dependent | 4.24 | 1.73E-04 |
| Fetal replicating | iPSC-derived cortical neurons | GO:0051254~positive regulation of RNA metabolic process | 4.21 | 1.75E-04 |
| Fetal replicating | iPSC-derived cortical neurons | GO:0045941~positive regulation of transcription | 4.19 | 2.04E-04 |
| Fetal replicating | iPSC-derived cortical neurons | GO:0016481~negative regulation of transcription | 4.17 | 1.60E-04 |
| Fetal replicating | iPSC-derived cortical neurons | GO:0000279~M phase | 4.17 | 1.03E-03 |
| Fetal replicating | iPSC-derived cortical neurons | GO:0003700~transcription factor activity | 4.15 | 9.47E-08 |
| Fetal replicating | iPSC-derived cortical neurons | GO:0010628~positive regulation of gene expression | 4.11 | 1.39E-04 |
| Fetal replicating | iPSC-derived cortical neurons | GO:0051301~cell division | 4.04 | 2.82E-03 |
| Fetal replicating | iPSC-derived cortical neurons | GO:0022403~cell cycle phase | 3.99 | 2.65E-04 |
| Fetal replicating | iPSC-derived cortical neurons | GO:0000278~mitotic cell cycle | 3.98 | 4.70E-04 |
| Fetal replicating | iPSC-derived cortical neurons | GO:0045934~negative regulation of nucleobase, nucleoside, nucleotide and nucleic acid metabolic process | 3.90 | 1.48E-04 |
| Fetal replicating | iPSC-derived cortical neurons | GO:0051172~negative regulation of nitrogen compound metabolic process | 3.88 | 1.44E-04 |
| Fetal replicating | iPSC-derived cortical neurons | GO:0051173~positive regulation of nitrogen compound metabolic process | 3.86 | 1.13E-04 |
| Fetal replicating | iPSC-derived cortical neurons | GO:0031328~positive regulation of cellular biosynthetic process | 3.80 | 1.08E-04 |
| Fetal replicating | iPSC-derived cortical neurons | GO:0045935~positive regulation of nucleobase, nucleoside, nucleotide and nucleic acid metabolic process | 3.79 | 1.60E-04 |
| Fetal replicating | iPSC-derived cortical neurons | GO:0010629~negative regulation of gene expression | 3.76 | 2.49E-04 |
| Fetal replicating | iPSC-derived cortical neurons | GO:0009891~positive regulation of biosynthetic process | 3.74 | 1.09E-04 |
| Fetal replicating | iPSC-derived cortical neurons | GO:0010557~positive regulation of macromolecule biosynthetic process | 3.74 | 1.46E-04 |
| Fetal replicating | iPSC-derived cortical neurons | GO:0010558~negative regulation of macromolecule biosynthetic process | 3.68 | 2.16E-04 |
| Fetal replicating | iPSC-derived cortical neurons | GO:0031327~negative regulation of cellular biosynthetic process | 3.65 | 2.34E-04 |
| Fetal replicating | iPSC-derived cortical neurons | GO:0009890~negative regulation of biosynthetic process | 3.58 | 2.52E-04 |
| Fetal replicating | iPSC-derived cortical neurons | activator | 3.51 | 3.03E-03 |
| Fetal replicating | iPSC-derived cortical neurons | GO:0051726~regulation of cell cycle | 3.39 | 4.08E-02 |
| Fetal replicating | iPSC-derived cortical neurons | GO:0006357~regulation of transcription from RNA polymerase II promoter | 3.19 | 5.36E-04 |
| Fetal replicating | iPSC-derived cortical neurons | GO:0022402~cell cycle process | 3.19 | 8.36E-04 |
| Fetal replicating | iPSC-derived cortical neurons | GO:0010604~positive regulation of macromolecule metabolic process | 3.18 | 1.83E-04 |
| Fetal replicating | iPSC-derived cortical neurons | developmental protein | 3.16 | 1.52E-02 |
| Fetal replicating | iPSC-derived cortical neurons | GO:0010605~negative regulation of macromolecule metabolic process | 2.95 | 8.15E-04 |
| Fetal replicating | iPSC-derived cortical neurons | GO:0015630~microtubule cytoskeleton | 2.87 | 1.51E-02 |
| Fetal replicating | iPSC-derived cortical neurons | GO:0007049~cell cycle | 2.82 | 5.96E-04 |
| Fetal replicating | iPSC-derived cortical neurons | GO:0005654~nucleoplasm | 2.73 | 8.82E-04 |
| Fetal replicating | iPSC-derived cortical neurons | GO:0030528~transcription regulator activity | 2.68 | 1.59E-05 |
| Fetal replicating | iPSC-derived cortical neurons | GO:0044430~cytoskeletal part | 2.52 | 1.68E-02 |
| Fetal replicating | iPSC-derived cortical neurons | GO:0005730~nucleolus | 2.49 | 2.27E-02 |
| Fetal replicating | iPSC-derived cortical neurons | GO:0031981~nuclear lumen | 2.32 | 4.32E-04 |
| Fetal replicating | iPSC-derived cortical neurons | GO:0051252~regulation of RNA metabolic process | 2.12 | 1.52E-03 |
| Fetal replicating | iPSC-derived cortical neurons | dna-binding | 2.12 | 6.01E-03 |
| Fetal replicating | iPSC-derived cortical neurons | GO:0006355~regulation of transcription, DNA-dependent | 2.11 | 2.27E-03 |
| Fetal replicating | iPSC-derived cortical neurons | transcription regulation | 1.98 | 6.67E-03 |
| Fetal replicating | iPSC-derived cortical neurons | Transcription | 1.91 | 1.00E-02 |
| Fetal replicating | iPSC-derived cortical neurons | GO:0070013~intracellular organelle lumen | 1.90 | 9.66E-03 |
| Fetal replicating | iPSC-derived cortical neurons | GO:0003677~DNA binding | 1.87 | 5.85E-03 |
| Fetal replicating | iPSC-derived cortical neurons | GO:0043233~organelle lumen | 1.87 | 9.60E-03 |
| Fetal replicating | iPSC-derived cortical neurons | GO:0031974~membrane-enclosed lumen | 1.84 | 1.08E-02 |
| Fetal replicating | iPSC-derived cortical neurons | GO:0045449~regulation of transcription | 1.75 | 6.43E-03 |
| Fetal replicating | iPSC-derived cortical neurons | nucleus | 1.72 | 1.38E-04 |
| Fetal replicating | iPSC-derived cortical neurons | GO:0043232~intracellular non-membrane-bounded organelle | 1.63 | 2.65E-02 |
| Fetal replicating | iPSC-derived cortical neurons | GO:0043228~non-membrane-bounded organelle | 1.63 | 2.65E-02 |
| Fetal replicating | iPSC-derived cortical neurons | phosphoprotein | 1.32 | 7.49E-03 |

**Supplementary table 3** **Biomark primers.** Pcw = post conceptional weeks

| **Target** | **Function** | **Forward primer** | **Reverse primer** | **Exon-exon spanning** |
| --- | --- | --- | --- | --- |
| ACTB | control | CCAACCGCGAGAAGATGAC | TAGCACAGCCTGGATAGCAA | TRUE |
| ADAMTS3 | occipital lobe (8-12 pcw) | CCTGGGGCTAGACATGTGTTAA | GGCCTGTAGCCTGGTTCTTAA | TRUE |
| AKR1C3 | layer 6b | AGCTGGGTTCCGCCATATA | GGTCGATGAAAAGTGGACCAA | TRUE |
| ANK2 | adaptor protein | TGGATGTGGCATCAGTCCTA | ACATGCAGGGGAGTAAAACC | TRUE |
| ANXA1 | layer 6b | AAGTGCGCCACAAGCAAA | TGCCTTATGGCGAGTTCCA | TRUE |
| B3GALT2 | layer 6 | TGATGGCGTGCCAAAAGTA | GTCACATTGTCTCTCTTGTAGTCA | TRUE |
| BCL11B | deep-layer neurons | CAACCCGCAGCACTTGTC | CCTCGTCTTCTTCGAGGATGG | TRUE |
| NDNF | layer 1 | CCAGATGGAGCTGAAATTAGCA | CTCCAAAGGCGCATCACA | TRUE |
| CACNA1E | layer 2 | GCCTTTGAAGCTCGTGTCA | AAGAGGTACCATGGCCTTCA | TRUE |
| CARTPT | layer 2 | TGAGAAGAAGTATGGCCAAGTCC | ATCCTTGCCCCTTTCCTCAC | TRUE |
| CBLN1 | hindbrain (cerebellum and upper rhombic lip) (all) | GCGCAAAGGGATCTACAGTTTTAA | CCAGCGAAGGCTGAAATCAC | TRUE |
| CBLN2 | frontal lobe (all) | TCAGCTTCCACGTGGTCAAA | AGGCCGAGATCACTGGGTA | TRUE |
| CCK | layer 2 | AGAACGGATGGCGAGTCC | ACGATGGACATTCGTCCAGAA | TRUE |
| CDH24 | layer 6b | TTTTCCCCTTGGGCCCTAC | GTGAGCAGTCACCTGGATCA | TRUE |
| CHRNA3 | layer 4 | AGACCAACCTGTGGCTCAA | ATGAACTCTGCCCCACCATA | TRUE |
| CHRNA7 | layer 1 | CCCAGATGGCCAGATTTGGAA | AGTGTGGAATGTGGCGTCAA | TRUE |
| CNR1 | layer 1 | ACGTCTGAGGATGGGAAGGTA | AGGACCAGGGTCTTGGCTAA | FALSE |
| CPNE7 | layer 5 | AGCCGAACGAGTACCTGAA | CAAACCCCAAAGCGGAAAAC | TRUE |
| CUX1 | progenitor cells and upper-layer neurons | ACGGACCTTGAAAGGGCAAA | CCGATGAGAGCTGTTCCCTTAA | TRUE |
| CYP26A1 | frontal lobe (8-12 pcw) | AGACCCTTCGACTGAATCCC | CAGCCCTTGGGAATCTGGTA | TRUE |
| DBH | adrenergic | GAGTGGGAGATCGTGAACCA | ACCGACACGACCTTCTTCAA | TRUE |
| DLG4 | postsynaptic marker | AGCTGGAGCAGGAGTTCAC | ACACGCTTCACCTTGTGGTA | TRUE |
| EBF3 | hindbrain (cerebellum and upper rhombic lip) (8-12 pcw) | CTCCTGGGCGCTTTGTCTA | CTTGGGATCACTTTCTGCAACC | TRUE |
| EOMES | secondary progenitor cells | CTGTGGCAAAGCCGACAATA | CTCATCCAGTGGGAACCAGTA | TRUE |
| FEZF2 | telencephalon (all) | CGCTCTGCAGGCACAAAATTA | GCCGCACTGGTTGCATTTA | TRUE |
| FOXG1 | telencephalon (all) | GCCAGCAGCACTTTGAGTTA | TGAGTCAACACGGAGCTGTA | FALSE |
| GABRA2 | GABA receptor | GCCAATCAATCGGAAAGGAGAC | TCAGGTGGAAATGAGCTGTCA | TRUE |
| GABRA5 | GABA receptor & layer 6 | GCTCTTGGATGGCTACGACAA | TGACGTAGATGTCGGTCCTCA | TRUE |
| GABRB1 | GABA receptor | TGGGGCTTCTCTCTTTCCC | ATGACATGTTGCTGGGTTCA | TRUE |
| GAD1 | GABA synthesis | ATCCTGGTTGACTGCAGAGAC | CCAGTGGAGAGCTGGTTGAA | TRUE |
| GAD2 | GABA synthesis | CTGCTCCAAAGTGGATGTCAAC | AAAGTGGGCCTTTCTCCATCA | TRUE |
| GAPDH | control | GAACGGGAAGCTTGTCATCAA | ATCGCCCCACTTGATTTTGG | TRUE |
| GFAP | glia | GCCAGTTGCAGTCCTTGAC | GCGCATCTGCCTCTCCA | TRUE |
| GPHN | inhibitory synapses | GTGGTTGCAGTCATGTCAACA | CGATTGCTGTCTCGAATCTTCC | TRUE |
| GRIA1 | AMPA receptor | CGCTCCACGTGATTGAAATGAA | TGGCTGCAGGGACAAACTTA | TRUE |
| GRIA2 | AMPA receptor | TGGAATGGGATGGTTGGAGAA | CACCTCTTCTCTCACAAGGGTA | TRUE |
| GRIA3 | AMPA receptor | TGGGGCTTTTGGGTCATTC | CTCCTGCACTGTGTTTCTCA | TRUE |
| GRIA4 | AMPA receptor | GCAAATCCTGCTGCTCCA | ATTCCCTGTCAGCCCTTGAA | TRUE |
| GRIN1 | NMDA receptor | GGCAACACCAACATCTGGAA | CCATCCGCATACTTGGAAGAC | TRUE |
| GRIN2A | NMDA receptor | GACCGGCCTCAGTGACAA | AGGCACTGTCCCAAATCGAA | TRUE |
| GRIN2B | NMDA receptor | TGCACCCGAAACTGGTGATA | TGCAGGGACTTGTCTTTCCA | TRUE |
| HNF1A | liver | TGGTACGTCCGCAAGCA | ACCTGTGGGCTCTTCAATCA | TRUE |
| HOXB2 | myelencephalon | GCACGGCTTACACCAACAC | GGCCGGCACAGGTACTTATTA | FALSE |
| HOXB3 | myelencephalon | CCCTTCGTCATGAATGGGATC | GGCAGGCGACAAATCTCC | TRUE |
| HTR2C | serotonergic & layer 5 | AGACTGAAGCAATCATGGTGAAC | TGCTACTGGGCTCACAGAAA | TRUE |
| KCNC1 | potassium channel | GCTCTTCGAGGACCCGTAC | AGACCAGGATGAAGAAGAGGGAA | TRUE |
| KCNC3 | potassium channel | TCTTCGAGGACCCCTACTC | GAAGGTGGTGATGGAGATGA | TRUE |
| KCNK2 | layer 6 | AATCAGTCACTGGGATTTGGGAA | TGCGTGGTGAGATGTTTCCA | TRUE |
| MAP2 | pan−neuronal marker | CAACGGAGAGCTGACCTCA | CTACAGCCTCAGCAGTGACTA | TRUE |
| MBP | glia | GAGCTCCAGACCATCCAAGAA | GGGAGGGTCTCTTCTGTGAC | FALSE |
| MET | temporal lobe (all) | TCCCCAATGACCTGCTGAAA | CTTTTCCAAGGACGGTTGAAGAA | TRUE |
| MFGE8 | layer 3 | ACCCAGCAGCAATGACGATA | TGCGTCACCACACCTGTTA | TRUE |
| MIOX | kidney | CAAAGACAAGGCCAGCTTCC | GCGTGTGCATGAGCTTGTA | TRUE |
| MKI67 | cycling cells | AGAGTAACGCGGAGTGTCA | CTTGACACACACATTGTCCTCA | TRUE |
| MPPED1 | neocortex & telencephalon (all) | GCCCTGCTGGAGAAATGGAA | GGGGACCCAGTCCAGGAA | TRUE |
| NANOG | stem cell marker | TGCCTTGCTTTGAAGCATCC | TTTCTTCAGGCCCACAAATCAC | FALSE |
| NCAM1 | pan−neuronal marker | CTCCCAGTCCATGTACCTTGAA | GGTTCCCCTCCCAAGTGTAC | TRUE |
| NEFL | thalamus (8-12 pcw) | ACGTGAAGATGGCTTTGGATA | TAGGCAGATCGGCCAAAGAC | TRUE |
| NES | neural progenitor cell marker | GCTGCGGGCTACTGAAAA | CTGAGCGATCTGGCTCTGTA | TRUE |
| NEUROD6 | neocortex & telencephalon (all) | GGAGACGATGCGACACTCA | ACAGACTCATCAAACGGTAGTGTTA | TRUE |
| NR2F1 | temporal lobe (8-12 pcw) | CAAAGCCATCGTGCTGTTCAC | GCTCCTCACGTACTCCTCCA | TRUE |
| NR2F2 | amygdala (all) | CTCAAGGCCATAGTCCTGTTCA | CAAGCTTTCCACATGGGCTAC | TRUE |
| NTNG2 | layer 6b | GTGCCACTGCAGGTTCC | GTCACCACATTCAGGAAGTCA | TRUE |
| OLIG2 | glia | CGGAGCGAGCTCCTCAAA | ATGGCCCCAGGGGAAGATA | TRUE |
| OPRK1 | layer 6b | CCTTGGAGGCACCAAAGTCA | GGTCCCACCAGGAGTAGTCA | TRUE |
| PAX6 | neural progenitor cell marker | CCCCACATATGCAGACACACA | GAACTGACACACCAGGGGAAA | TRUE |
| PDE1A | layer 6 | GCATACAGGGACAACAAACAAC | TCTCAAGGACAGAGCGATCA | TRUE |
| PDYN | layer 4 & striatum & ganglionic eminence | CAGGATGGTCCCAAACCTATCA | AGAAAGCTCTGGCATCTCTCC | TRUE |
| POU3F2 | progenitor cells and upper-layer neurons | CGGATCAAACTGGGATTTACCC | CGAGAACACGTTGCCATACA | FALSE |
| POU5F1 | stem cell marker | GGTATTCAGCCAAACGACCAT | CCGCAGCTTACACATGTTCT | FALSE |
| PPP1R1B | parietal lobe (8-12 pcw) | TCTCAAGTCGAAGAGACCCAAC | TGCAGGTGAGACTCAGCAA | TRUE |
| PRSS12 | layer 3 | GTTAGCATTGCCTGCTACCC | TTTCTCCATCCATCAGTCTGACA | TRUE |
| RASGRF2 | layer 2 | CAAGGACTAAAGGCAAGGATAGCA | TGTTCAGCATGGGGAGCAAA | TRUE |
| RORB | layer 4 | GGCAGACCCACACCTATGAA | ATTGTTGCCACAGTGCTTCC | TRUE |
| RSPO3 | hippocampus (8-12 pcw) | GGAATACATCGGCAGCCAAA | TCCTTGGCAGCCTTGACTAA | TRUE |
| RXFP1 | layer 6 | AGTCGAATTTCCCCACCAACA | AAACGGGTGAGGACGTTATTCA | TRUE |
| RXRG | striatum (8-12 pcw) | ATGCCTGCGAGCCATTGTA | TCGCAGAGTCTCCACCTCA | TRUE |
| SATB2 | upper layers and some layer 5 neurons & neocortex | TTTGCCAAAGTGGCTGCAAA | TTTCTGGGCTTGGGTTCTCC | TRUE |
| SCN4B | layer 3 | GAAGTGGACAACACAGTGACA | CGAGGAGCTCACGAGACA | TRUE |
| SIX3 | amygdala (all) | GGCCTCACTCCCACACAA | ATGCCGCTCGGTCCAA | TRUE |
| SLC17A6 | glutamate transport | TGGGGCTACATCATCACTCA | GAAGTATGGCAGCTCCGAAA | TRUE |
| SLC17A7 | glutamate transport | GCCATCTCCTTCCTGGTCCTA | GGCTATGTCCAGGTGGTTCAC | TRUE |
| SLC32A1 | GABA transport | AGGCTGGAACGTGACCAAC | GAGAAACAACCCCAGGTAGCC | TRUE |
| SOX2 | neural progenitor cell marker | CATGAAGGAGCACCCGGATTA | CGGGCAGCGTGTACTTATCC | FALSE |
| SV2C | layer 3 | GGACAGAATTGGGCGCTTAA | GTGCCGAACCAAAGGAAGAA | TRUE |
| SYN1 | presynaptic marker | GCAAGGACGGAAGGGATCA | TGTCTTCATCCTGGTGGTCAC | TRUE |
| SYNDIG1L | striatum (all) | CGGGACCAGGAGGATGAC | AGCGTGAGGAAGTTGTCTTCA | TRUE |
| SYT2 | parietal lobe (all) | TGCATCCTGGAGGCTAAGAA | CTGCATCAGGTGGATCTTCAC | TRUE |
| SYT6 | layer 6b | TCTGACCTGTCTCGGGAAAC | AGAACATGATCTCTCCCAAGTCC | TRUE |
| TBR1 | prelate, layer 1 and deep-layer neurons & telencephalon (8-12 pcw) | ACGAACAACAAAGGAGCTTCA | TGGTACTTGTGCAAGGACTGTA | TRUE |
| TCF7L2 | thalamus (all) | GCACCGTAGGACAAATCCC | TCCTGTCGTGATTGGGTACA | TRUE |
| TENM2 | occipital lobe (all) | TGGCAGGCAAGGATAGCA | TTGGCCTCGGATGAGAGAAA | TRUE |
| TPH1 | serotonergic | GCGGACTTGGCTATGAACTA | GTTCCCCAGGTCTTAATCTCC | TRUE |
| TTR | hippocampus (all) | CTGCCTTGCTGGACTGGTA | GCCACATTGATGGCAGGAC | TRUE |
| TUBB3 | pan−neuronal marker | GAGCGGATCAGCGTCTACTA | GGTTCCAGGTCCACCAGAA | TRUE |
| VAT1L | layer 5 | TCCAACATGGTAACTGGAGAGAC | GATGGGGTTCACCTTCTCCA | TRUE |

**Supplementary table 4 Immunofluorescence co-expression of layer markers.** The mean ± standard deviation of percentages of DAPI positive cells positive for different combinations of layer markers is reported. Five separate fields of view were counted for each layer marker combination in AH017-3 iPSC-derived cortical neurons 140 days post-neural induction.

| **Deep layer marker** | **Upper layer marker** | **Deep layer alone**  **(%)** | **Upper layer alone**  **(%)** | **Deep and upper layer (%)** |
| --- | --- | --- | --- | --- |
| TBR1 | BRN2 | 13.4 ± 2.6 | 21.2 ± 2.4 | 26.5 ± 3.3 |
| TBR1 | CUX1 | 17.9 ± 2.5 | 11.9 ± 4.6 | 15.4 ± 4.2 |
| TBR1 | SATB2 | 25.9 ± 8.0 | 39.0 ± 15.7 | 7.8 ± 2.8 |

**Supplementary table 5 Co-expression analysis of different combinations of canonical cortical layer markers.** Q-values are empirical 2-tailed measures of significance from 10,000 permutations of the single cell RT-qPCR data correcting for multiple hypothesis testing using the Benjamini-Hochberg method.

| **Marker 1** | **Marker 2** | **Correlation (r)** | **Significance (q)** |
| --- | --- | --- | --- |
| *BCL11B* | *TBR1* | 0.06 | 0.36 |
| *BCL11B* | *CUX1* | 0.14 | 0.02 |
| *BCL11B* | *POU3F2* | 0.23 | 0.001 |
| *BCL11B* | *SATB2* | -0.03 | 0.61 |
| *TBR1* | *CUX1* | -0.04 | 0.55 |
| *TBR1* | *POU3F2* | -0.01 | 0.86 |
| *TBR1* | *SATB2* | 0.08 | 0.26 |
| *CUX1* | *POU3F2* | 0.27 | < 0.0002 |
| *CUX1* | *SATB2* | 0.03 | 0.61 |
| *POU3F2* | *SATB2* | 0.14 | 0.03 |
